# Honey-fried licorice decoction ameliorates atrial fibrillation susceptibility by inhibiting the NOX2–ROS–TGF-β1 pathway

**DOI:** 10.3389/fphar.2025.1595111

**Published:** 2025-07-23

**Authors:** Yu Qin, Jingyan Wang, Rong Chen, Zhiling Tang, Hao Zhi, Xiang Wu, Xiaodong Chen, Jialei Fu, Xueting Cai, Jianping Shen, Peng Cao, Qian Zhou

**Affiliations:** ^1^ Affiliated Hospital of Integrated Traditional Chinese and Western Medicine, Nanjing University of Chinese Medicine, Nanjing, China; ^2^ State Key Laboratory of Materials-Oriented Chemical Engineering, College of Food Science and Light Industry, Nanjing Tech University, Nanjing, China; ^3^ Nanjing Drum Tower Hospital, Affiliated Hospital of Medical School, Nanjing University, Nanjing, China; ^4^ State Key Laboratory on Technologies for Chinese Medicine Pharmaceutical Process Control and Intelligent Manufacture, Nanjing University of Chinese Medicine, Nanjing, China

**Keywords:** honey-fried licorice decoction, atrial fibrillation, oxidative stress, NOX2-ROS-TGF-β1 pathway, fibrosis

## Abstract

**Objective:**

Honey-fried licorice decoction (HFLD), a well-established traditional Chinese medicine, is widely used to treat atrial fibrillation (AF) in China. However, the specific cardioprotective mechanisms of HFLD in treating AF remain unclear. This study aimed to determine the efficacy of HFLD and validate the efficacy and mechanisms of action of HFLD in reducing AF susceptibility.

**Methods:**

Serum oxidative stress biomarker levels of healthy controls and patients with paroxysmal AF were detected using enzyme-linked immunosorbent assay kits. The HFLD components were identified using ultra-performance liquid chromatography coupled with quadrupole time-of-flight mass spectrometry. Wistar rats were intraperitoneally injected with isoprenaline (5 mg/kg) for 2 weeks to construct an AF rat model. The effect of HFLD on AF was assessed with the transesophageal atrial pacing technique and histopathological analysis. The expression levels of NOX2-ROS-TGF-β1 signaling pathway related proteins were detected using Western blot and dihydroethidium staining.

**Results:**

Our clinical trial verified that the expression of MDA increased, whereas SOD, CAT and GSH/GSSG ratio decreased in the serum of patients with paroxysmal AF compared with that in individuals with a normal sinus rhythm. Notably, HFLD treatment could reverse these imbalances. In rat experiments, HFLD was found to reduce oxidative stress and extracellular matrix deposition, thereby effectively reducing AF’s induction rate and duration. Western blot analysis indicated that HFLD downregulated the expression of NOX2 and its regulatory proteins, leading to the inhibition of the downstream TGF-β1–SMAD3 signaling pathway.

**Conclusion:**

HFLD may reduce AF susceptibility by inhibiting the NOX2–ROS–TGF-β1 pathway, potentially providing new perspectives on AF treatment.

## 1 Introduction

Atrial fibrillation (AF) is the most prevalent type of supraventricular arrhythmia, affecting an estimated 33.5 million people worldwide (Hu D. et al., 2023). AF reduces cardiac output, increases the risk of atrial thrombus formation, and may lead to cardiac failure, arterial embolization, and stroke, resulting in a significant socioeconomic burden (Wiczer et al., 2017). Recently, there have been substantial advancements in the management of AF. However, current antiarrhythmic drugs are reportedly associated with organ-toxicity profiles and a risk for proarrhythmia (Saleh and Haldar, 2023; Zhou Q. et al., 2024). Furthermore, catheter ablation is limited by a high recurrence rate (Benz et al., 2024).

Traditional Chinese medicine (TCM) is reportedly effective in alleviating clinical symptoms in patients with AF, with fewer adverse effects than those associated with current antiarrhythmic drugs (Jiang et al., 2022; Shi et al., 2023; Zhang et al., 2018; Zhou T. et al., 2024). Honey-fried licorice decoction (HFLD) is a TCM prescription for treating severe palpitations and irregular pulses according to the Treatise on Febrile Diseases (Shang-Han-Lun). Based on Chinese medicine theory, the main effects of HFLD include promoting Yang, restoring the veins, benefiting Qi, and nourishing Yin. Therefore, HFLD is typically prescribed to treat cardiovascular diseases because of a deficiency in both Yin and Yang. Scientific research has shown that HFLD exerts a protective effect against diabetic myocardial infarction by decreasing inflammation and inhibiting cardiomyocyte apoptosis (Hu M. et al., 2023). A meta-analysis including 39 clinical trials revealed that HFLD combined with metoprolol (Met) demonstrated improved efficacy and minimal adverse reactions in managing arrhythmia (Yang et al., 2022). Additionally, primary data have indicated that HFLD might be effective in treating AF by reversing atrial electrical remodeling and myocardial fibrosis (Guo et al., 2023; Sun et al., 2019). However, the specific mechanisms of action of HFLD in treating AF are still not fully understood.

NADPH oxidases (NOXs) comprise the major source of reactive oxygen species (ROS) in the cardiovascular system. Oxidative stress derived from NOXs contributes to paroxysmal atrial fibrillation (PAF) by causing electrophysiological changes and structural remodeling of the atrium (Ramos-Mondragón et al., 2023; Liu X. et al., 2023; Yoo et al., 2020). Notably, the expression of NOX2, a key isoform of NOXs expressed in the heart (Sciarretta et al., 2014; Frantz et al., 2006), is reportedly upregulated in patients with PAF (Cangemi et al., 2012). ROS produced in atrial tissues play key roles in atrial remodeling and fibrosis (Zhou et al., 2022) and participate in modulating transforming growth factor-beta 1 (TGF-β1) signaling through several pathways, especially the SMAD pathway (Liu and Desai, 2015). The TGF-β–SMAD pathway is the most important activating signal for cardiac fibrosis (Zeng et al., 2019), of which TGF-β1 is a key mediator of fibrosis development and exerts its biological effects by activating downstream mediators SMAD family member 3 (SMAD3) (Hu et al., 2018). ROS-induced TGF-β1 upregulation has been reported to be vital in the progression of cardiovascular diseases, including AF (Yang et al., 2020; Lin et al., 2022; Garlapati et al., 2023). However, whether HFLD decreases AF susceptibility by inhibiting the NOX2–ROS–TGF-β1 pathway remains unclear.

Thus, in the current study, our objectives were to assess the levels of oxidative stress in patients with PAF, determine the efficacy of HFLD, characterize the chemical compositions of HFLD for quality control, and evaluate the efficacy and mechanisms of action of HFLD in reducing AF susceptibility in rats.

## 2 Materials and methods

### 2.1 Patients

The clinical trial was approved by the Ethics Committee of Jiangsu Province Academy of Traditional Chinese Medicine (2022-LWKY-025) and has been registered at the Chinese Clinical Trial Registry (ChiCTR2400085614). Informed consent was obtained from all participants. According to the guidelines (Hindricks et al., 2021), PAF is defined as AF that resolves spontaneously or is terminated by intervention within 7 days of onset. Thirty-one adult patients diagnosed with PAF based on 12-lead or 24-h ambulatory electrocardiograph (ECG) reports at our institution between July 2023 and October 2023 were included in this study. Exclusion criteria included: patients with history of coronary artery disease, valvular heart disease, chronic renal failure, hyperthyroidism, cerebrovascular accident and ongoing systemic inflammation (such as in the case of infection, cancer, rheumatoid arthritis or chronic obstructive pulmonary disease) (Li et al., 2017). In addition, six of the patients with PAF were treated with HFLD for 4 weeks. The HFLD granules were provided by Jiangyin Tianjiang Pharmaceutical (China). TCM syndrome scores (see detailed evaluation method in Supplementary Table S1) and sera were examined at baseline and at the end of treatment. Thirty-three healthy volunteers without a history of arrhythmia were included in the normal sinus rhythm (NSR) group. The collected sera were stored at −80°C until use for expression analysis.

### 2.2 HFLD preparation

According to Shang-Han-Lun, HFLD is composed of Glycyrrhizae Radix Et Rhizoma Praeparata Cum Melle (also named honey-fried licorice, Zhigancao in Chinese, 12 g), Zingiberis Rhizoma Recens (Shengjiang in Chinese, 9 g), Ginseng Radix et Rhizoma (Renshen in Chinese, 6 g), Rhemanniae Radix (Dihuang in Chinese, 48 g), Cinnamoni Ramulus (Guizhi in Chinese, 9 g), Asini Corii Colla (Ejiao in Chinese, 6 g), Ophiopogonis Radix (Maidong in Chinese, 10 g), Cannabis Fructus (Maren in Chinese, 10 g), and Jujubae Fructus (Dazao in Chinese, 25 g). All herbs were purchased from the Dispensary of TCM of the Jiangsu Provincial Hospital on Integration of Chinese and Western Medicine.

HFLD was mixed and immersed in 1,400 mL 8% glutinous rice wine and 1,600 mL distilled water for 0.5 h. The compounds were extracted for 1 h with reflux. The extracted solution was filtered through gauze, and the procedure was repeated. Both extracts were combined, added to melted Ejiao, and concentrated to 3.1 g/mL for rats experiments. The remainder solution was concentrated and then made into a lyophilized powder for ultra-performance liquid chromatography coupled with quadrupole time-of-flight mass spectrometry (UPLC-QTOF/MS).

### 2.3 UPLC-QTOF/MS instrumentation and conditions

For UPLC, an Acquity HSS T3 chromatographic column (Waters; 2.1 × 150 mm, 1.8 μm) was used. UPLC was performed in conjunction with a SCIEX Exion LC instrument and an X500B Q-TOF mass spectrometer (AB SCIEX, United States). The injection volume was 2 μL, and the flow rate was 0.3 mL/min. The mobile phase was divided into 0.01% aqueous formic acid solution (phase A) and acetonitrile (phase B). Separation was achieved using the following gradient program: 3%–8% B at 0–5 min, 8%–30% B at 5–11 min, 30%–80% B at 11–20 min, 80%–95% B at 20–21 min, 95% B at 21–27 min, and 3% B at 27.5–34 min. The column and autosampler temperature were maintained at 35°C and 4°C, respectively. Standard substances were used to identify the components of the HFLD.

### 2.4 Animal experiments

Sixty adult male Wistar rats weighing 250 ± 20 g were purchased from the Experimental Animal Business Department of the Shanghai Institute of Planned Parenthood Research (China). All animals were reared in a room under 12 h light/dark cycles with a temperature of 22°C–24°C and a relative humidity of 40%–60% and had free access to food and water. All animal experiments were approved by the Committee of Experimental Animals of Jiangsu Province Academy of Traditional Chinese Medicine (AEWC-20221015-242) and complied with the National Institutes of Health’s Guidelines for the Care and Use of Laboratory Animals.

**FIGURE 1:**
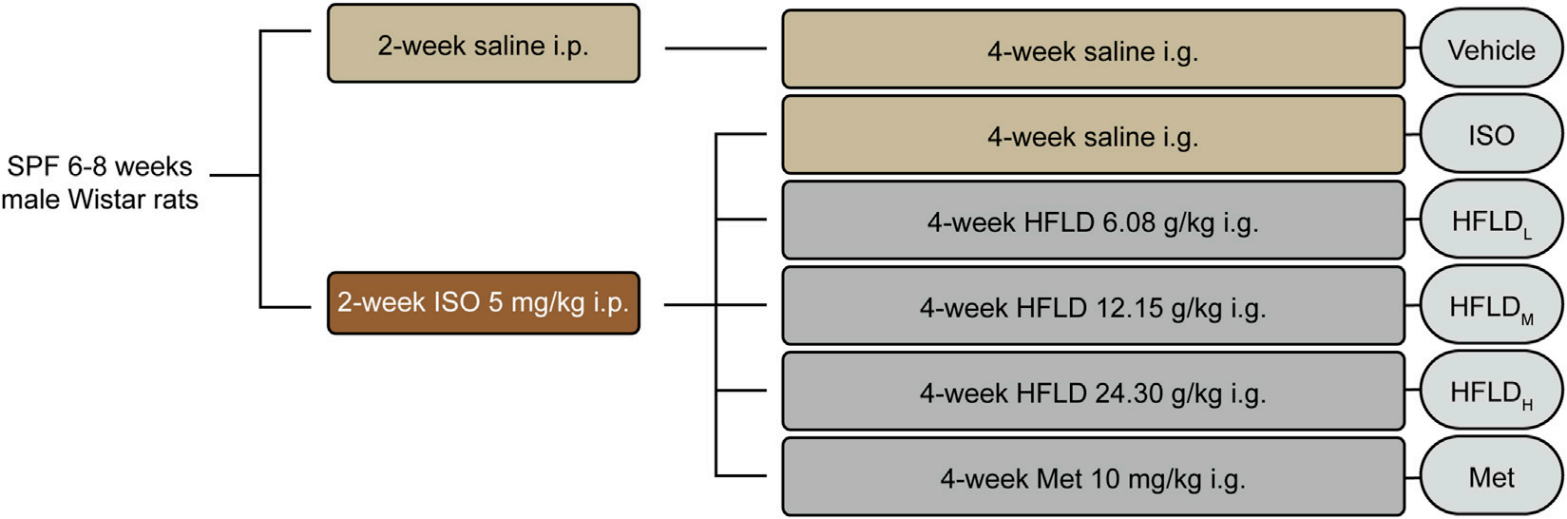
Schematic experimental procedure for HFLD treatment of the rat model.

As shown in Figure 1, the rats were randomly divided into two groups after 1 week of adaptive feeding. The rats in the experimental group (*n* = 52) were intraperitoneally injected with isoprenaline hydrochloride (ISO; 5 mg kg^–1^·d^–1^; TargetMol; United States; T1056) for 2 weeks (Wei et al., 2020), whereas those in the vehicle group (*n* = 8) underwent equal treatments of normal saline. On day 14, The ECGs were monitored using PowerLab monitoring systems and LabChart 8 software (ADInstruments, Australia) to identify drug-sensitive rats. Later, 40 rats in the experimental group were randomly assigned to five groups and gavaged for 4 weeks with saline (ISO group, *n* = 8), a low dose of HFLD (6.08 g kg^–1^·d^–1^; HFLDL group, *n* = 8), moderate dose of HFLD (12.15 g kg^–1^·d^–1^; human-equivalent dosage; HFLDM group, *n* = 8), high dose of HFLD (24.30 g kg^–1^·d^–1^; HFLDH group, *n* = 8), or Met (10 mg kg^–1^·d^–1^ Met tartrate tablets; Met group, *n* = 8) (Fowler et al., 2018). The same volume of saline was administered to the vehicle group for 4 weeks. The rats were euthanized after atrial stimulation, and their sera and hearts were collected for further research.

### 2.5 Echocardiography

The rats were initially anesthetized with 4.5% isoflurane, after which, the concentration was maintained at 1.5%–2%. Echocardiography was conducted using a VINNO 6 LAB ultrasound system (VINNO, China). Two-dimensional guide M-mode echocardiographic data were recorded. Each echocardiographic variable was determined from at least three cardiac cycles.

### 2.6 ECG analysis

Before injection, at the time of the last ISO injection, and on the day after the last gavage, the rats were anesthetized and then implanted with subcutaneous electrodes to monitor ECGs.

For atrial stimulation, a 6-French quadripolar catheter was guided through the esophagus and positioned at the site with the lowest threshold for atrial capture. A 50 Hz burst pacing was applied at 3 V above the diastolic threshold for 3 s to assess the susceptibility to AF (Luo et al., 2022; Halațiu et al., 2022). Five successive stimulation cycles were applied to each rat. The onset of AF was indicated by the disappearance of P waves, an irregular R–R interval, and the appearance of tiny f waves, whereas its termination was marked by the recovery of P waves and sinus rhythm, and the disappearance of tiny f waves. We recorded total AF inductions and total AF durations.

### 2.7 Enzyme-linked immunosorbent assay (ELISA) analysis

The serum levels of creatine kinase-MB (CK-MB; SCIBEN, AE4147), lactate dehydrogenase (LDH; SCIBEN, AE4093), N-terminal pro-brain natriuretic peptide (NT-proBNP; SCIBEN, AE4148), matrix metalloproteinase-9 (MMP-9; SCIBEN, AE4152), interleukin-6 (IL-6; SCIBEN, AE4149 and AE0048), and tumor necrosis factor-alpha (TNF-α; SCIBEN, AE4087 and AE0120) were assessed using commercial ELISA kits according to the manufacturer’s protocols.

### 2.8 Biochemical analysis

Human sera and rat-myocardial tissue homogenates were prepared, and the malondialdehyde (MDA; Beyotime, S0131), superoxide dismutase (SOD; Beyotime, S0101), catalase (CAT; Servicebio, G4307) and glutathione/glutathione disulfide (GSH/GSSG; Servicebio, G4304) contents were measured according to the manufacturer’s instructions. Protein concentrations of tissue homogenates were determined using the BCA Protein Assay Kit (Beyotime, P0012).

### 2.9 Morphological and histological analyses

Histopathological analyses of atrial tissues were performed to observe the effect of HFLD on the structural remodeling in rats. Heart sections were dehydrated, embedded in paraffin, cut into 4-μm-thick sections using a microtome, and mounted on slides. After dewaxing, Masson trichrome staining (Servicebio, G1006) were performed to reveal fibrotic areas. Briefly, the sections were dehydrated by being treated with gradient ethanol one by one and then washed with tap water. Subsequently, they were immersed in potassium dichromate overnight, and then dyed in a haematoxylin solution for 1 min. Further, the sections were incubated with ponceau and acid fuchsin dye for 6 min, phosphomolybdic acid aqueous solution for 1 min, and aniline blue dye for 30 s. Finally, the slides were treated with 1% acetic acid solution, dehydrated, mounted with mounting solution, and observed under ECLIPSE E100 microscope (Nikon, Japan).

For immunohistochemical staining, the heart sections were incubated with antibodies against collagen I (Servicebio, GB124197-100) and collagen III (Servicebio, GB115674-100) overnight at 4°C. After incubation, the sections were washed gently in phosphate-buffered saline (PBS) and incubated with horseradish peroxide secondary antibodies for 50 min. The sections were then subjected to DAB colorization followed by hematoxylin restaining. Finally, a microscope was utilized to observe the tissue sections.

The cross-sectional areas of the cardiomyocytes were observed via fluorescein-conjugated wheat germ agglutini (WGA) staining. Briefly, paraffin sections were subjected to EDTA antigen repair solution (Servicebio, G1206) at 100°C for 20 min and returned to room temperature naturally, then washed with PBS. The sections were stained with sufficient staining solution (Sigma Aldrich, L4895) and incubated at 37°C in the dark for 1 h. PBS was rinsed three times again. Nuclei were stained with DAPI. After sealing the slide with cover glass, the images were observed and collected by ECLIPSE Ci fluorescence microscopy (Nikon, Japan) in the dark room. The positive areas were quantified using ImageJ software (National Institutes of Health, United States).

### 2.10 Dihydroethidium (DHE) staining

DHE staining was used to assay the production of ROS O_2_^•−^ *in situ* (Ren et al., 2019). Briefly, atrial tissue slides were incubated with DHE (Sigma Aldrich, D7008) at 37°C for 30 min, then washed, fixed, mounted, and subjected to fluorescence microscopy.

### 2.11 Western blot analysis

Atrial tissues were dissected and homogenized in RIPA buffer (Beyotime, P0013) containing protease and phosphatase inhibitors (Thermo Fisher Scientific, A32959). Proteins were separated via 8%–15% sodium dodecyl sulfate-polyacrylamide gel electrophoresis and then transferred to a polyvinylidene fluoride membrane. The membranes were blocked with Tris-buffered saline with Tween (TBST) containing 5% bovine serum albumin for 1 h and then incubated with a primary antibody at 4°C overnight. On the following day, the membranes were washed thrice with TBST, incubated with the secondary antibody for 1.5 h, and washed again. Protein bands were then visualized using an enhanced chemiluminescence reagent (Thermo Fisher Scientific, United States; 34580) and an automated 5200 chemiluminescent imaging system (Tanon, China). Glyceraldehyde-3-phosphate dehydrogenase (GAPDH) was used as an internal control, and the protein bands were analyzed using ImageJ. The antibodies used for Western blot analysis are listed in Supplementary Table S2.

### 2.12 Statistical analysis

The data are expressed as the mean ± standard error of the mean (SEM) and analyzed using GraphPad Prism software (version 9.0). Comparisons of categorical variables between two groups were performed using the chi-square test, differences in continuous variables between two groups were compared using Student’s t-test. Differences among the six groups were analyzed using one-way analysis of variance (ANOVA) after confirming the assumptions of normality (Shapiro-Wilk test) and homogeneity of variance (Brown-Forsythe test). Following a significant one-way ANOVA, Tukey’s *post hoc* test was performed to assess pairwise differences among all six groups. Adjusted *P*-values are reported to account for multiple comparisons. *P* < 0.05 was considered to reflect a statistically significant difference.

## 3 Results

### 3.1 Characteristics of the study participants

The clinical manifestations observed in the NSR and PAF groups were remarkably similar (Table 1), indicating that these samples were statistically qualified for a comparative study.

**TABLE 1:** Characteristics of patients with NSR and PAF.

Demographic/clinical characteristics	NSR (*n* = 33)	PAF (*n* = 31)	*P* value
Age, years*	65.27 ± 2.01	68.71 ± 1.75	0.20
Male, *n* (%)	14 (42.42)	16 (51.61)	0.62
Heart rate, beats/min	75.94 ± 1.74	76.32 ± 3.83	0.93
Hypertension (BP > 139/89 mmHg), *n* (%)	22 (66.67)	24 (77.42)	0.41
Diabetes mellitus, *n* (%)	6 (18.18)	8 (25.81)	0.55
Hyperlipidemia, *n* (%)	6 (18.18)	6 (19.35)	>0.99
Carotis artery lesion, *n* (%)	11 (33.33)	9 (29.03)	0.79
Beta-blocker use, *n* (%)	6 (18.18)	7 (22.58)	0.76
Statin use, *n* (%)	6 (18.18)	7 (22.58)	0.76

*All values are expressed as the mean ± standard error of the mean. Age distribution and heart rate were analyzed using Student’s t-test, and the remaining indicators were analyzed using the chi-squared test. NSR, normal sinus rhythm; PAF, paroxysmal atrial fibrillation; BP, blood pressure.

### 3.2 HFLD treated PAF by alleviating oxidative stress and inflammation

**FIGURE 2:**
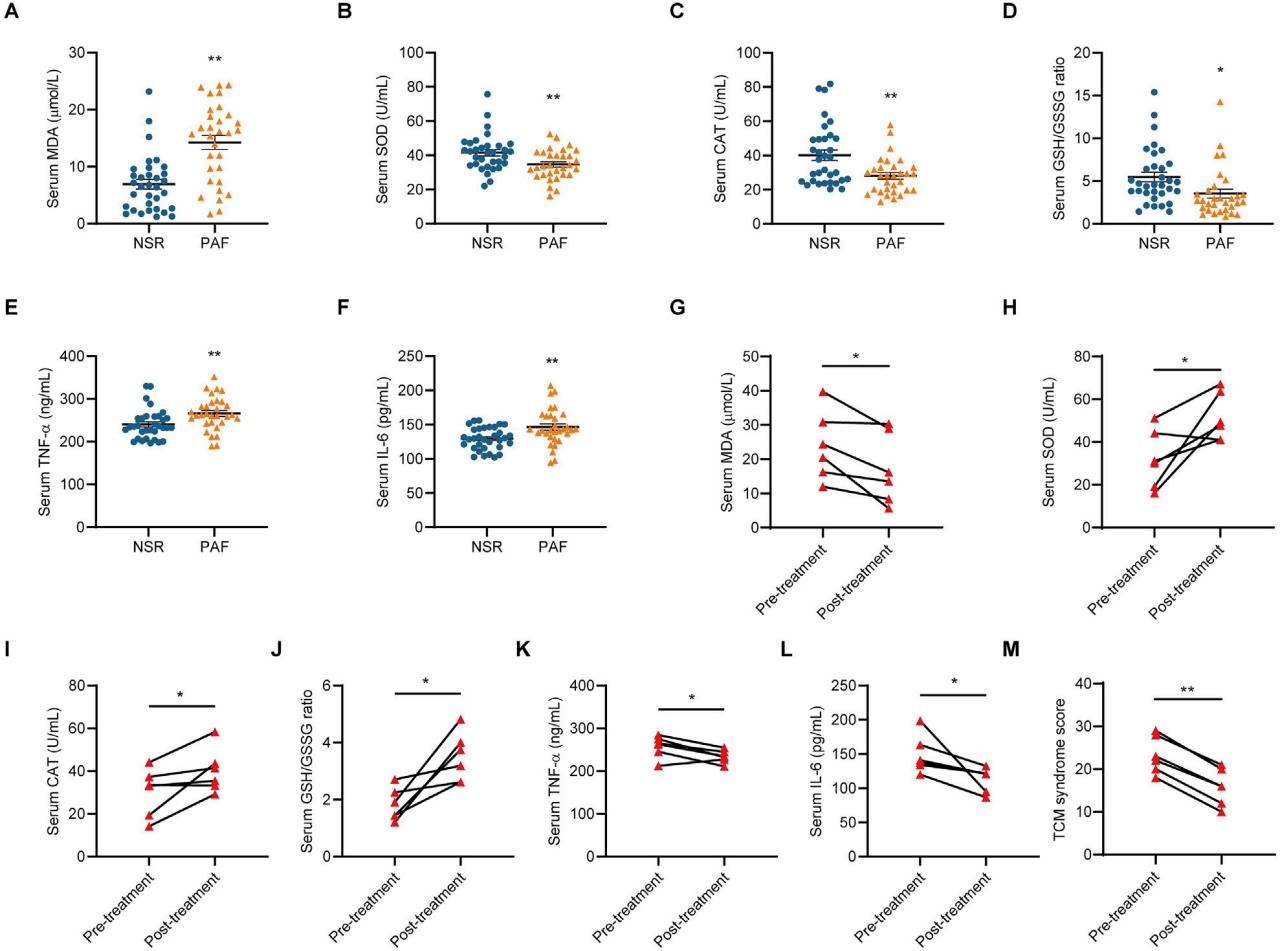
HFLD treated PAF by alleviating oxidative stress and inflammation. **(A–F)** The levels of MDA, SOD, CAT, GSH/GSSG ratio, TNF-α and IL-6 in serum samples from patients in the NSR and PAF groups. NSR, *n* = 33; PAF, *n* = 31. Compared using unpaired Student’s t-test. ^*^ *P* < 0.05, ^**^ *P* < 0.01 compared with the NSR group. **(G–M)** The changes in serum MDA, SOD, CAT, GSH/GSSG ratio, TNF-α, IL-6 and TCM syndrome scores in patients with PAF between the pre-treatment and post 28 days HFLD treatment (*n* = 6). Compared using paired Student’s t-test. ^*^ *P* < 0.05, ^**^ *P* < 0.01 compared with pre-treatment.

While evaluating the expression levels of oxidative stress markers and key inflammatory cytokines, it was observed that patients with PAF exhibited higher MDA, TNF-α and IL-6 contents (Figures 2A,E,F), but lower SOD, CAT activities and GSH/GSSG ratio (Figures 2B–D), than individuals with NSR. However, following a 4-week HFLD treatment, these indicators showed improvements (Figures 2G–L). In addition, TCM syndrome scores decreased in patients with PAF between the pre-treatment and post-28 days HFLD treatment (Figure 2M). Importantly, no recurrence of AF was observed during the whole HFLD-treatment period and the follow-up period. These results indicated that oxidative stress was involved in the progression of PAF and that HFLD could prevent AF recurrence and alleviate clinical symptoms related to PAF by inhibiting oxidative stress.

### 3.3 UPLC-QTOF/MS-based detection of the main components of HFLD

**FIGURE 3:**
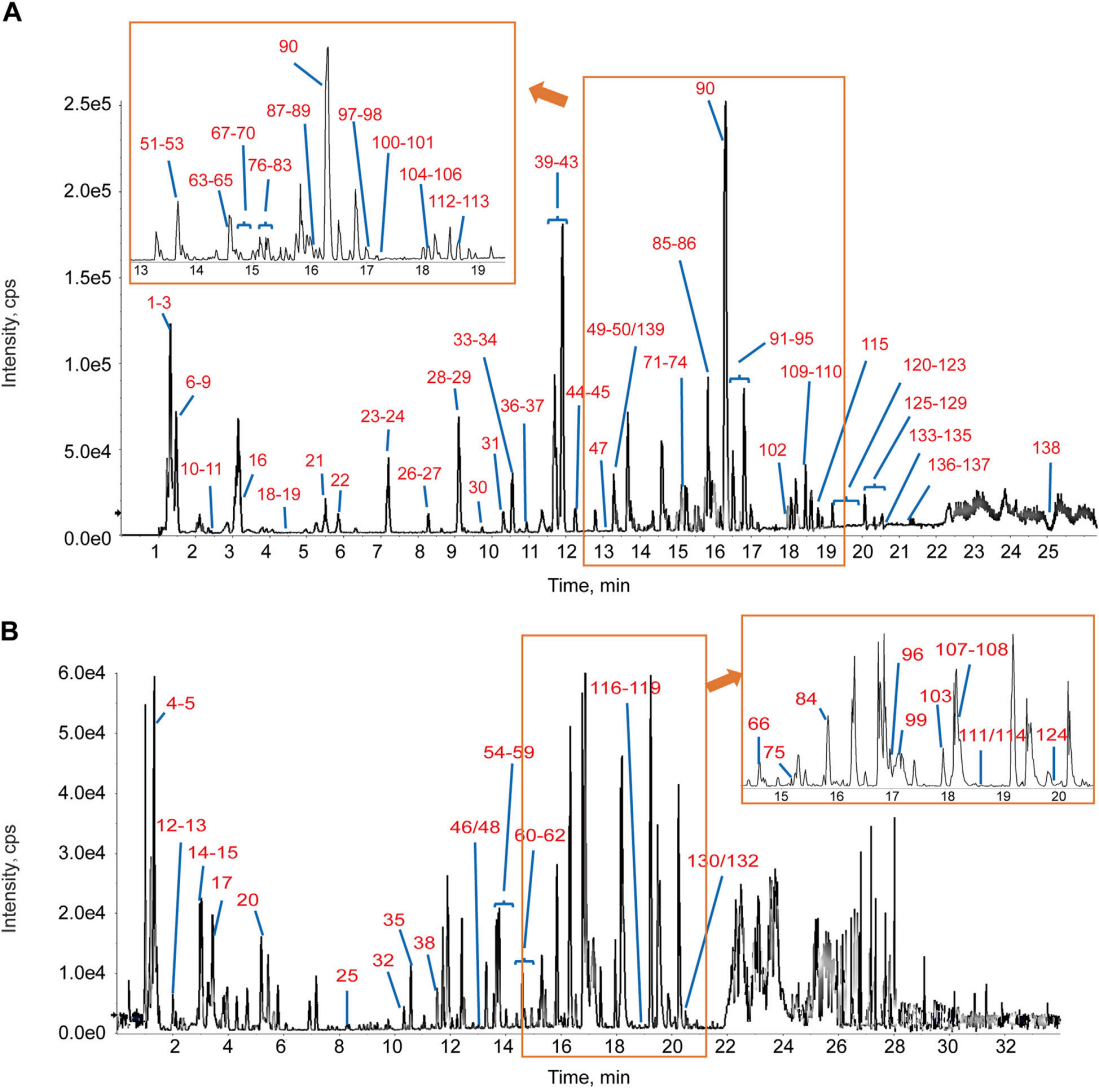
Base-peak intensity chromatograms of HFLD using ultra-performance liquid chromatography coupled with quadrupole time-of-flight mass spectrometry. **(A)** Negative-ion mode. **(B)** Positive-ion mode. The peak number corresponds to the compound number in Supplementary Table S3.

The base-peak ion chromatograms of both electrospray ionization modes (positive and negative) were obtained using UPLC-QTOF/MS (Figure 3A). A total of 139 components were identified based on the molecular formula, MS/MS-fragmentation behavior, and comparison with reference standards, including flavonoids, flavonoid glycosides, saponins, sugars, organic acids, coumarins, amino acids, and iridoid glycosides. The identities and MS/MS data of these compounds were obtained, and most ingredients originated from honey-fried licorice and Ginseng Radix et Rhizoma (Supplementary Table S3).

### 3.4 ISO treatment led to myocardial damage in rats

Rats in the experimental group were intraperitoneally injected with ISO (5 mg/kg) for 2 weeks, and rats in the vehicle group were injected with the same volume of normal saline (Figure 1).

**FIGURE 4:**
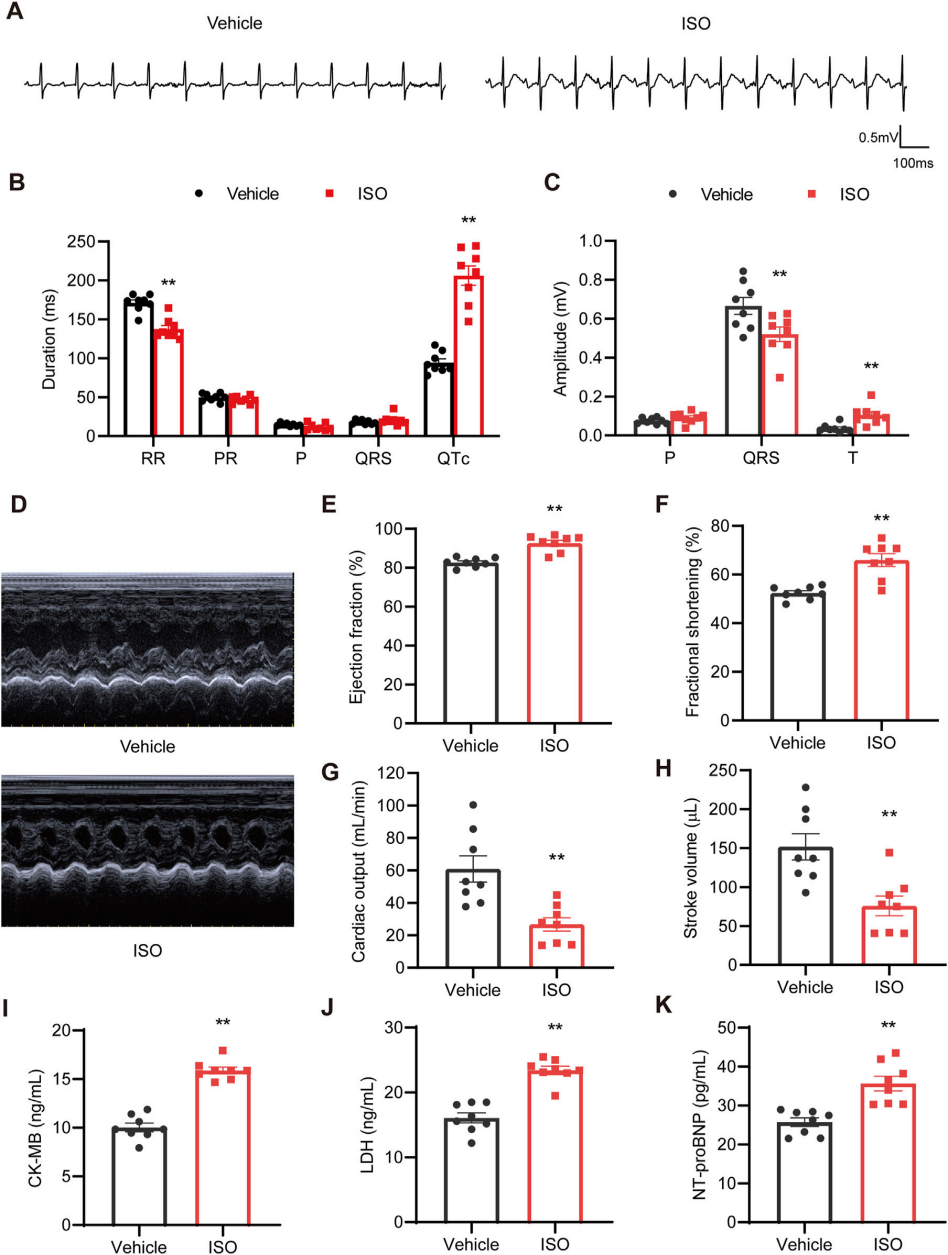
ISO caused myocardial damage in rats. **(A)** Representative ECG tracings on day 14. **(B)** RR, PR, P wave, QRS, and QTc duration quantification (*n* = 8). **(C)** P wave, QRS, and T wave amplitude quantification (*n* = 8). **(D)** Representative M-mode images of echocardiography. **(E–H)** Statistical analyses of the ejection fraction, fractional shortening, cardiac output, and stroke volume (*n* = 8). **(I–K)** Serum levels of CK-MB, LDH, and NT-proBNP (*n* = 8). ^*^ *P* < 0.05, ^**^ *P* < 0.01 compared with the vehicle group.

There were notable changes in ECG morphology in rats of the ISO group (Figure 4A). Specifically, the RR interval was shortened and QTc prolonged in the ISO group compared to those in the vehicle group (Figure 4B). Meanwhile, the QRS amplitude decreased and the ST segment was significantly elevated (Figure 4C).

Cardiac structure and function were assessed using echocardiography (Figure 4D). Consistent with previous results (Li et al., 2021), echocardiographic examinations indicated a compensatory increase in ejection fraction (EF, Figure 4E) and fractional shortening (FS, Figure 4F). Additionally, ISO treatment significantly reduced cardiac output (Figure 4G) and stroke volumes (Figure 4H). Furthermore, ISO triggered increased interventricular septum and left-ventricular posterior wall thickness, along with a reduction in the left-ventricular internal diameter (Supplementary Table S4).

In addition, elevated levels of myocardial injury markers, including CK-MB, LDH, and NT-proBNP, were observed in rats following ISO treatment (Figures 4I–K). These findings suggest that ISO application significantly induced cardiac hypertrophy and established cardiac injury in rats, which could effectively increase their susceptibility to AF in rats (Ma et al., 2020).

### 3.5 HFLD reduced AF susceptibility in rats

**FIGURE 5:**
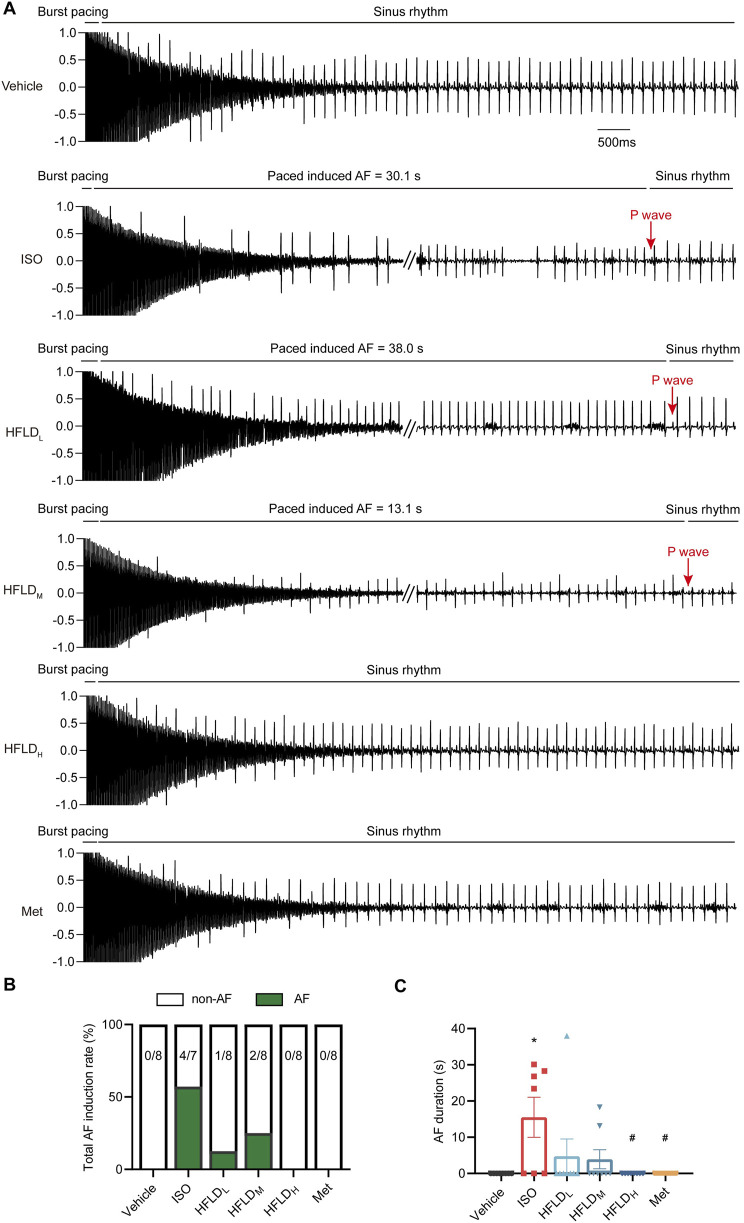
HFLD decreased the susceptibility to AF in rats 4 weeks after ISO injection. **(A)** Representative ECG recordings after atrial-burst pacing. **(B)** Total induction rates of AF in each group. **(C)** Total AF durations in each group. ^*^ *P* < 0.05 compared with the vehicle group. ^#^ *P* < 0.05 compared with the ISO group.

Rats were then assigned into six groups and treated with normal saline, HFLD with different concentrations, or Met, respectively. One hour after the last treatment, programmed electrical stimulation was performed to detect the susceptibility to AF (Figure 5A). Normal saline did not cause AF in rats in the vehicle group, as the induction rate was 0 (0/8); one rat in the ISO group died during stimulation, so the total induction rate in the ISO group was 57.14% (4/7). HFLD or Met treatment effectively decreased the induction rate (Figure 5B). Additionally, the total AF duration significantly increased under ISO injection, whereas it was reduced after the HFLD and Met treatments (Figure 5C).

### 3.6 HFLD prevented ISO-induced myocardial damage in rats

Post-treatment evaluation via ECG and echocardiography revealed no significant alterations in most cardiac parameters. However, a decline in EF and FS was observed, suggesting mild systolic dysfunction. HFLD and Met interventions partially mitigated these deteriorations, suggesting a potentially beneficial role in preserving cardiac performance (Supplementary Tables S5, S6).

**FIGURE 6:**
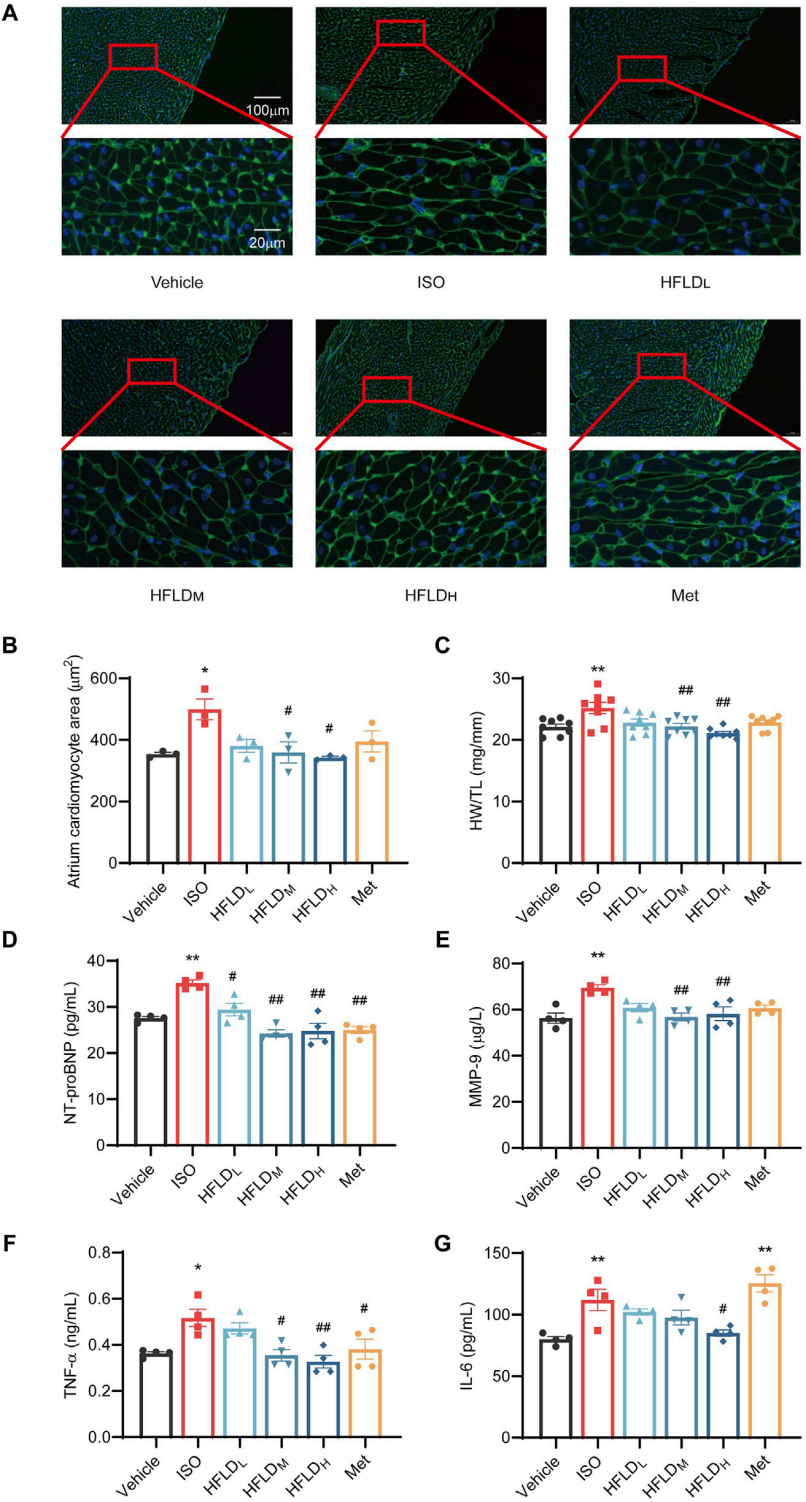
HFLD prevented ISO-induced myocardial damage in rats. **(A)** Representative WGA staining (×20 and ×100 magnification; scale bar: 100 μm and 20 μm). **(B)** Quantification data of WGA staining (*n* = 3). **(C)** HW: TL ratio in each group (*n* = 8). **(D–G)** Levels of serum NT-proBNP, MMP-9, TNF-α, and IL-6 (*n* = 4).^*^ *P* < 0.05, ^**^ *P* < 0.01 compared with the vehicle group. ^#^ *P* < 0.05, ^##^ *P* < 0.01 compared with the ISO group.

Moreover, our results showed that compared with the vehicle group, ISO induced an increased cardiomyocyte size, and HFLD treatment attenuated the ISO-induced hypertrophy in cardiomyocytes in a concentration-dependent manner (Figures 6A,B). Moreover, the heart weight (HW): tibia length (TL) ratio increased in the ISO group, which was significantly prevented in HFLD-fed rats (Figure 6C). Met also alleviated these trends to some extent but the efficacy was not as good as that of HFLD. These results indicate that HFLD significantly suppressed cardiac hypertrophy in ISO-induced cardiac injury.

The vasoactive peptide, NT-proBNP, is a crucial biomarker of AF (Bai et al., 2023). MMP-9 and its associated tissue inhibitor of metalloproteinases are key molecules for tissue remodeling (Kaireviciute et al., 2010). Therefore, we measured serum NT-proBNP, MMP-9, TNF-α, and IL-6 levels. We observed that the ISO group displayed markedly higher levels of these biomarkers than the vehicle group, and HFLD treatment significantly mitigated this cytokine release caused by myocardial damage (Figures 6D–G). Notably, HFLD_H_ was able to attenuate these biomarkers to a level similar to that in the vehicle group. Based on these results, HFLD was shown to be protective against ISO-induced myocardial injury in rats.

### 3.7 HFLD suppressed oxidative stress of atrial tissues by blocking the activation of NOX2 signaling

**FIGURE 7:**
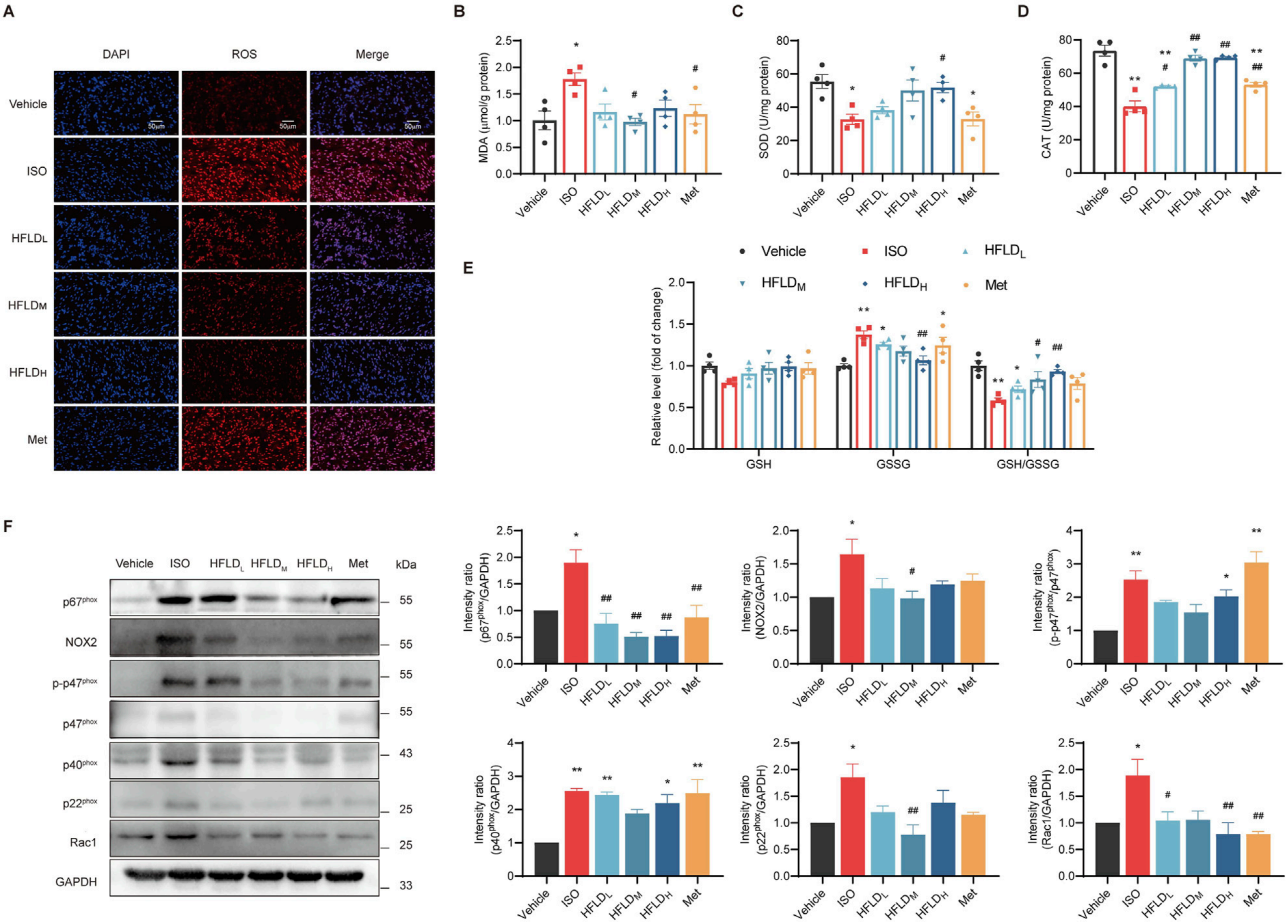
HFLD suppressed oxidative stress by blocking the activation of NOX2 signaling in rats. **(A)** Representative dihydroethidium staining performed to analyze ROS production in the atrium (×40 magnification; scale bars: 50 μm). **(B)** MDA contents of rat atrial tissues (*n* = 4). **(C)** SOD activities in atrial tissues (*n* = 4). **(D)** The activities of CAT in rat atrial tissues (*n* = 4). **(E)** The relative concentrations of GSH, GSSG and GSH/GSSG ratio in rat atrial tissues (*n* = 4). **(F)** Western blot analysis of NOX2 and its subunits protein expression (*n* = 3). ^*^ *P* < 0.05, ^**^ *P* < 0.01 compared with the vehicle group. ^#^ *P* < 0.05, ^##^ *P* < 0.01 compared with the ISO group.

ISO also causes oxidative stress (Liao et al., 2022). Therefore, we detected the expression of oxidative stress markers in atrial tissues. Compared with the vehicle group, significantly higher DHE fluorescence was observed in the ISO group, indicating that ISO successfully led to ROS production. Meanwhile, HFLD treatment attenuated the expression of ROS (Figure 7A). Furthermore, the activities of SOD, CAT and GSH/GSSG ratio were remarkably decreased, and MDA concentrations increased in atrial tissues after ISO treatment. These alterations were partially reversed by HFLD (Figures 7B–E), indicating that HFLD contributed to free-radical scavenging.

The expression levels of NOX2 and its subunits were further examined with Western blot analysis (Figure 7F). ISO treatment resulted in significantly higher protein expression of NOX2, p67^phox^, p-p47^phox^, p40^phox^, p22^phox^, and Rac1 than vehicle treatment. However, these ISO-induced changes were significantly inhibited by HFLD treatment. These results suggest that HFLD demonstrated cardioprotective effects by alleviating oxidative stress and inhibiting the NOX2 signaling pathway.

### 3.8 HFLD reduced cardiac fibrosis of atrial tissues by regulating the TGF-β1–SMAD3 pathway

**FIGURE 8:**
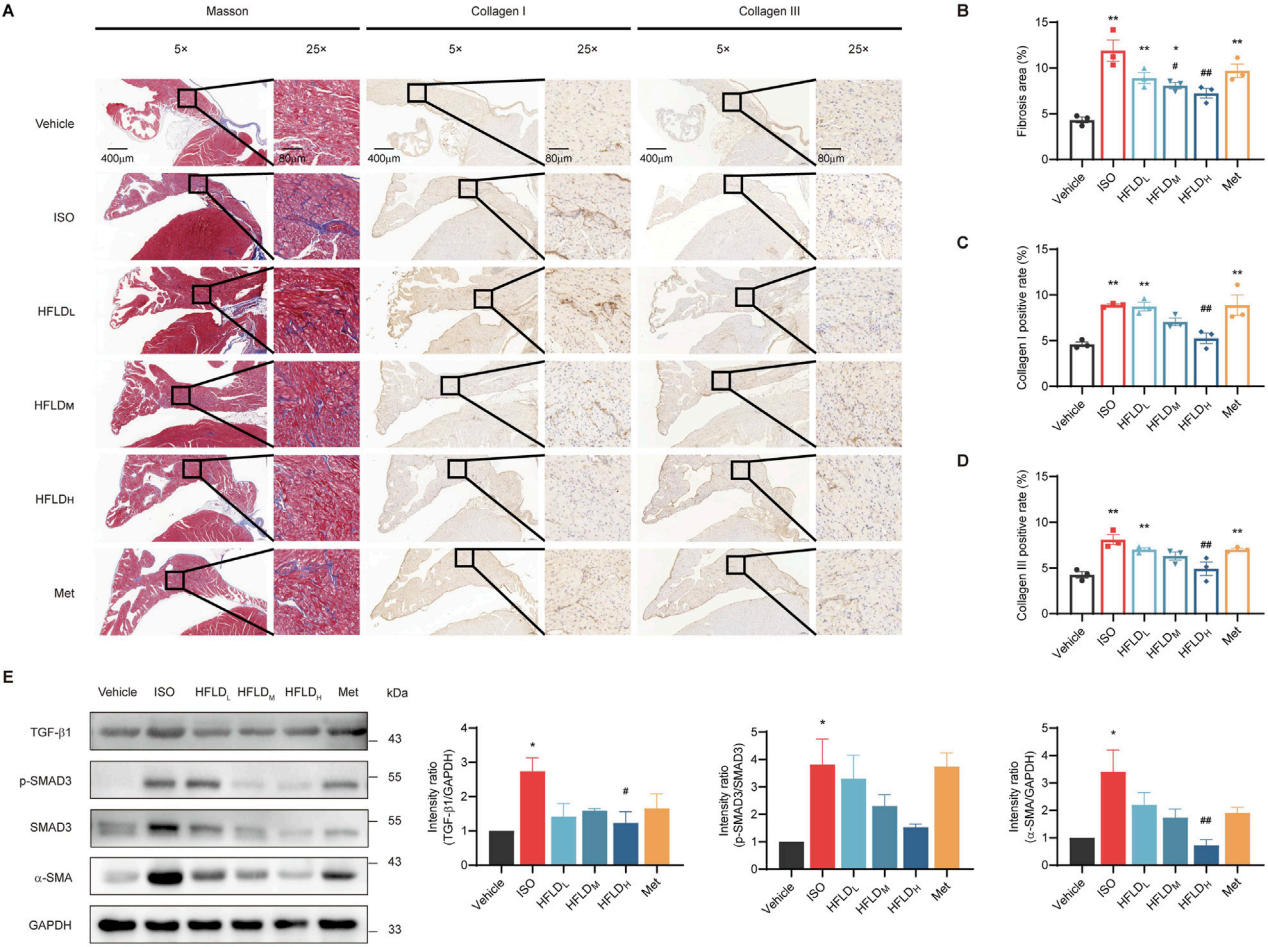
HFLD reduced atrial fibrosis by regulating the TGF-β1–SMAD3 pathway in rats. **(A)** Masson’s trichrome staining and immunochemistry revealed collagen I and collagen III expression in atrial tissues (×5 and ×25 magnification; scale bars: 400 μm and 80 μm). **(B–D)** Quantitative data of fibrosis and collagen I and collagen III expressions (*n* = 3). **(E)** Western blot analysis of TGF-β1, p-SMAD3, SMAD3, and α-SMA protein expression (*n* = 3). ^*^ *P* < 0.05, ^**^ *P* < 0.01 compared with the vehicle group. ^#^ *P* < 0.05, ^##^ *P* < 0.01 compared with the ISO group.

Oxidative stress is known to be involved in cardiac fibrosis (Liu Z-Y. et al., 2023). To further study the mechanism through which HFLD blocks myocardial injury, we first detected atrial fibrosis in rats via Masson’s trichrome staining and immunohistochemistry and the relevant results are shown in Figure 8A. Compared to the vehicle group, ISO induced obvious atrial fibrosis, and HFLD reduced the ISO-induced atrial fibrosis. The semi-analysis further supported our observations, showing that the atrial fibrosis areas in the ISO group were markedly larger than those in the vehicle group (Figures 8B–D). In addition, HFLD therapy decreased the positive areas in a dose-dependent manner, and positive drug Met attenuated the ISO-induced atrial fibrosis similarly to HFLD_L_.

We further detected TGF-β1, p-SMAD3, and alpha-smooth muscle actin (α-SMA) expression via Western blot analysis (Figure 8E). Our results validated that ISO significantly increased the expression of TGF-β1, p-SMAD3, and α-SMA compared to the respective expressions in the vehicle group, and treatment with HFLD suppressed these expression. In contrast, these protein expression levels in the HFLD_H_ group decreased more significantly compared with those in other groups. These results suggest that HFLD attenuated atrial fibrosis by regulating the TGF-β1–SMAD3 pathway.

## 4 Discussion

HFLD has been widely used in China for cardiovascular diseases through supplementing Yang and nourishing Ying and was believed to be effective and safe (Wang et al., 2022; Tsai et al., 2017). Although previous studies of HFLD have reported the possible mechanism underlying AF treatment (Guo et al., 2023; Sun et al., 2019), few clinical trials have been designed and conducted rigorously. Large-scale, randomized, placebo-controlled clinical trials are still warranted to investigate the detailed mechanism of action of HFLD.

Oxidative stress involves a state of disequilibrium between endogenous antioxidant defenses and ROS production (van der Pol et al., 2019) and plays a major role in the homeostasis and function of the cardiovascular system, especially in the pathophysiology of AF and associated complications (Yang et al., 2020; Li et al., 2017). Consistent with previous findings (Li et al., 2017; Wu et al., 2023), our results revealed that the contents of MDA increased and the activities of SOD, CAT, and the GSH/GSSG ratio decreased in patients with PAF compared to those in NSR controls, further confirming the implication of oxidative stress in the progression of AF. Furthermore, the expression of key inflammatory cytokines, including TNF-α and IL-6, were assessed, revealing significantly elevated serum levels of both TNF-α and IL-6 in patients with PAF. Although the number of subjects was small, our clinical trial findings suggest that HFLD significantly increased circulating SOD, CAT, and GSH/GSSG ratio and decreased MDA, TNF-α and IL-6 levels, indicating that HFLD may treat AF by alleviating oxidative stress and inflammation.

HFLD is composed of nine medicinal substances and 139 active ingredients with various components that target multiple pathways, potentially explaining its broad effects on AF. For example, honey-fried licorice, the principal drug in HFLD, has demonstrated protective effects against arrhythmia and can mitigate oxidative stress and tissue damage induced by arrhythmia. Moreover, studies have further confirmed that liquiritigenin, licoflavonol, and isoliquiritigenin are the active constituents responsible for honey-fried licorice’s efficacy in treating arrhythmia (Wang et al., 2025). To further confirm the efficacy outcome of HFLD in treating AF, a rat model was established and the therapeutic effect of HFLD was investigated. ISO reportedly increases myocardial hypoxia, resulting in increased ROS production, which further exacerbates oxidative stress (Ren et al., 2019). Rats with ISO-induced AF showed altered oxidative stress levels, which facilitated inflammation responses and impaired normal cardiac function. ROS can lead to excessive MDA production and cell-structure damage. Antioxidant enzymes, such as SOD and CAT, are crucial for ameliorating free radical-induced oxidative damage and maintaining balanced oxidation and antioxidation processes in the body (Khatua et al., 2012). Our results confirm that ISO led to increased myocardial ROS production and that HFLD intervention resulted in lower MDA and ROS levels and higher SOD and CAT activities than those induced by ISO treatment. Additionally, the GSH/GSSG ratio serves as an important indicator of cellular redox state. Compared to the vehicle group, the ISO group displayed a significantly lower GSH/GSSG ratio, demonstrating elevated oxidative stress and diminished antioxidant capacity in atrial. Our findings suggest that HFLD might protect against antioxidative defenses by effectively neutralizing ROS and their related metabolites induced by ISO. Therefore, we focused on the antioxidative activity of HFLD to further investigate the mechanisms underlying AF treatment.

Phagocytic NOX consists of a multicomponent complex involving transmembrane flavocytochrome b558, which is a heterodimeric assembly of NOX2 and p22^phox^ and supported by the cytosolic protein factors p40^phox^, p47^phox^, p67^phox^, and small GTP-binding proteins (Rac1 or Rac2). In a physiological state, these diverse components physically dissociate into an inactive state. When activated, the regulatory subunits assemble with the flavocytochrome b558 in the cell membrane (Vermot et al., 2021). The phosphorylation and translocation of p47^phox^ are vital in the activation of enzyme complexes. The upregulation of NOX2 and translocation of p47^phox^ and Rac1 to the cell membrane are the primary regulatory factors leading to increased NOX2 activity (Ren et al., 2019). In this study, HFLD treatment resulted in significantly lower NOX2 protein levels in myocardial tissues than did ISO treatment. Furthermore, HFLD downregulated p47 expression and phosphorylation and reduced the upregulation of p22^phox^, p40^phox^, p67^phox^, and Rac1, indicating that HFLD inhibited NOX2 activity by transcriptionally and post-translationally regulating the expression of NOX2 subunits.

Previous basic experimental and clinical trial data showed that ROS levels are related to atrial tissue remodeling (Yang et al., 2020) and that the upregulation of ROS activated TGF-β1 (Wu et al., 2022; Zhang et al., 2019). TGF-β1 signaling is considered a “master” signaling pathway that participates in fibroblast activation and extracellular matrix production. Targeted regulation of TGF-β–SMAD signaling pathways may represent a new mechanistic approach for counteracting ISO-induced cardiac fibrosis. TGF-β1 mediates canonical signaling by binding its receptor in the plasma membrane and inducing phosphorylation of the SMAD2 and SMAD3 transcription factors. As a result, phosphorylated SMAD2 and SMAD3 interact with SMAD4 in the cytoplasm, which subsequently translocates to the nucleus and stimulates the transcription of fibrosis-related genes, such as collagen I and collagen III (Zeng et al., 2019). In this study, we observed extensive deposition of collagen I and collagen III in the atrial tissues of rats in the ISO group. Meanwhile, TGF-β1 and p-SMAD3 production was significantly upregulated, showing that the TGF-β1–SMAD3 pathway participated in ISO-induced atrial fibrosis. HFLD treatment improved heart functions and inhibited TGF-β1 and p-SMAD3 production. TGF-β1 can stimulate myofibroblast activation based on α-SMA expression (Pedron et al., 2010). Cardiac fibroblasts, the most abundant interstitial cells in the adult mammalian heart, are the main source of collagen I and collagen III. In pathological conditions, injurious factors cause cardiac fibroblasts to differentiate into cardiac myofibroblasts and express higher levels of α-SMA, collagen Ⅰ, and collagen III, thereby contributing to the progression of cardiac fibrosis (Wang et al., 2023). Our results revealed that HFLD prevented cardiac fibroblasts from differentiating into myofibroblasts. In conclusion, the results of our rat experiments demonstrate that HFLD eliminated oxidative stress, thereby attenuating cardiac fibrosis and delaying the occurrence and progression of AF by inhibiting the NOX2–ROS–TGF-β1 pathway.

However, this study has several limitations. Firstly, although our clinical trial was well-designed and performed to explore the benefits of HFLD, the number of included volunteers was small, representing a limited demographic, which may not suffice to widely generalize the findings. The specific criteria for patient selection and the narrow time frame might limit the diversity of the sample, which could affect the applicability of the results to other populations with different genetic, environmental, or lifestyle factors. Studies including more diverse patient cohorts are essential to validate the translatability of the results. Secondly, while our study demonstrates the efficacy of HFLD in reducing AF susceptibility, it is important to acknowledge that the use of different formulations between decoction and granule phases may introduce variability in drug bioavailability. Future studies could directly compare the two formulations to further validate translational consistency. Additionally, oxidative stress can activate multiple downstream intracellular signaling pathways, such as the NOX2–ROS–p38 pathway (Yin et al., 2015) and the NOX2–ROS–NF-κB pathway (Zhao et al., 2017), which further exacerbates pathological changes in myocardial tissues. Future research including NOX2-overexpression and NOX2-knockout experiments are necessary to definitively establish the relationship between NOX2 and TGF-β1 pathways. Further research is necessary to determine whether HFLD impacts downstream signaling pathways related to oxidative stress.

## 5 Conclusion

In conclusion, this study provides a detailed insight into the mechanism of action of HFLD in reducing AF susceptibility. Our clinical trial suggests that HFLD decreased the TCM syndrome scores of patients with PAF by alleviating oxidative stress, providing fundamental support for further exploring the interaction between oxidative stress and AF susceptibility. Our animal experiments suggested that HFLD reduced oxidative stress and ameliorated atrial pathological damage, thereby reducing AF susceptibility probably by regulating the NOX2–ROS–TGF-β1 pathway. While NOX2 emerges as a promising therapeutic target by which HFLD reduces AF susceptibility, this will be confirmed in further studies.

## Data Availability

The original contributions presented in the study are included in the article/Supplementary Material, further inquiries can be directed to the corresponding authors.
