# Supplementary material for: Honey-fried licorice decoction ameliorates atrial fibrillation susceptibility by inhibiting the NOX2–ROS–TGF-β1 pathway
Source: Front Pharmacol. 2025 Jul 23;16:1595111. doi: 10.3389/fphar.2025.1595111 (PMC12325216; doi:10.3389/fphar.2025.1595111)
Supplement: Supplementary file 1 [file Table1.docx]

Supplementary Material

**Supplementary Table 1.** The quantitative classification criteria of TCM syndrome score

|  | TCM syndrome score | | | |
| --- | --- | --- | --- | --- |
| **Primary symptoms** | 0 | 2 | 4 | 6 |
| Palpitation | No | Occasional palpitations, the discomfort is slight | Marked palpitations, the duration is longer, and the discomfort is obvious | Severe palpitations, difficult to calm down, and even affect daily life |
| Shortness of breath | No | Shortness of breath after ordinary physical activities | Shortness of breath after less-than-ordinary physical activities | Shortness of breath even while at rest |
| Lassitude | No | Mental lassitude of essence-spirit, but can continue ordinary physical activities | General lassitude can barely adhere ordinary physical activities | Severe mental and physical lassitude difficult to carry out daily activities |
| **Secondary symptoms** | 0 | 1 | 2 | 3 |
| Dizziness | No | Mildly | Moderately | Severely |
| Chest stuffiness | No | Mildly | Moderately | Severely |
| Chest pain | No | Mildly | Moderately | Severely |
| Spontaneous day perspiration | No | Mildly | Moderately | Severely |
| Night perspiration | No | Mildly | Moderately | Severely |
| Dry mouth | No | Mildly | Moderately | Severely |
| Bitter taste | No | Mildly | Moderately | Severely |
| Insomnia | No | Mildly | Moderately | Severely |
| Lusterless complexion | No | Mildly | Moderately | Severely |
| Vexing heat in the chest, palms and soles | No | Mildly | Moderately | Severely |
| Amaurosis | No | Occasionally | Sometimes | Often |

According to the Guidance Principle of Clinical Studies on New Traditional Chinese Medicine Drug (in Chinese), published by Ministry of Health, People's Republic of China.

**Supplementary Table 2.** Antibodies used for western blot analysis

| Resource | Manufacturer | Cat No. | Dilution |
| --- | --- | --- | --- |
| p67^phox^ | ABclonal | A3703 | 1:2000 |
| NOX2 | ABclonal | A1636 | 1:1000 |
| p-p47^phox^ | Thermo | PA5-36863 | 1:500 |
| p47^phox^ | ABclonal | A1148 | 1:1000 |
| p40^phox^ | ABclonal | A2096 | 1:1000 |
| Rac1/2/3 | ABclonal | A7720 | 1:1000 |
| p22^phox^ | Cell Signaling Technology | 37570S | 1:1000 |
| TGF-β1 | Proteintech | 21898 | 1:2000 |
| p-SMAD3 | Cell Signaling Technology | 9520T | 1:1000 |
| SMAD3 | ABclonal | A19115 | 1:5000 |
| α-SMA | ABclonal | A17910 | 1:2000 |
| GAPDH | Cell Signaling Technology | 5174S | 1:2000 |
| Goat anti-Rabbit IgG (H+L)-HRP | Bioworld | BS13278 | 1:50000 |

**Supplementary Table 3.** Characterization of chemical constituents of HFLD by UPLC-QTOF/MS

| **No.** | **Identification** | **Formula** | **RT(min)** | **Ion Mode** | **Calc *m/z*** | **Exact *m/z*** | **ppm** | **Fragment ions(*m/z*)** | **Source** |
| --- | --- | --- | --- | --- | --- | --- | --- | --- | --- |
| 1 | L-Arginine | C_6_H_14_N_4_O_2_ | 1.22 | [M+H]^+^ | 175.1195 | 175.1181 | -7.99 | 130, 70, 60 | O |
| 2 | Raffinose | C_18_H_32_O_16_ | 1.23 | [M-H]^-^ | 503.1618 | 503.1616 | -0.40 | 383, 281, 179, 101, 89 | R |
| 3 | Glucose | C_6_H_12_O_6_ | 1.28 | [M-H]^-^ | 179.0561 | 179.0565 | 2.18 | 89, 71, 59 | J |
|  |  |  |  | [M+Na]^+^ | 203.0526 | 203.0530 | 1.92 | - |  |
| 4 | Stachyose | C_24_H_42_O_21_ | 1.31 | [M+Na]^+^ | 689.2111 | 689.2122 | 1.63 | 527, 345 | R/J |
| 5 | Sucrose/Melibiose/Gentiobiose | C_12_H_22_O_11_ | 1.37 | [M-H]^-^ | 341.1089 | 341.1097 | 2.35 | 131, 119, 89, 71, 59 | C/J/R/GR |
|  |  |  |  | [M+NH_4_]^+^ | 360.1506 | 360.1504 | -0.56 | 145, 127, 85 |  |
| 6 | Inulin | C_36_H_62_O_31_ | 1.39 | [M-H]^-^ | 989.3202 | 989.3210 | 0.78 | 827, 341 | J |
| 7 | Fructose//Galactose | C_6_H_12_O_6_ | 1.40 | [M-H]^-^ | 179.0556 | 179.0565 | 5.03 | 89, 71, 59 | R |
| 8 | Verbascose | C_30_H_52_O_26_ | 1.40 | [M+Cl]^-^ | 863.2435 | 863.2456 | 2.43 | 827 | R |
| 9 | Adenine | C_5_H_5_N_5_ | 1.42 | [M-H]^-^ | 134.0472 | 134.0475 | 2.24 | 134, 107, 65 | G/C |
|  |  |  |  | [M+H]^+^ | 136.0623 | 136.0613 | -7.35 | 119, 92, 67 |  |
| 10 | Citric acid | C_6_H_8_O_7_ | 2.34 | [M-H]^-^ | 191.0197 | 191.0201 | 1.94 | 111, 87, 85 | C |
| 11 | cAMP | C_10_H_12_N_5_O_6_P | 2.49 | [M-H]^-^ | 328.0453 | 328.0463 | 3.20 | 134 | J |
|  |  |  |  | [M+H]^+^ | 330.0598 | 330.0605 | 2.12 | 136 |  |
| 12 | Uracil | C_4_H_4_N_2_O_2_ | 2.69 | [M+H]^+^ | 113.0351 | 113.0344 | -6.19 | 96, 95, 70, 68, 44, 42, 40 | G |
| 13 | Uridine | C_9_H_12_N_2_O_6_ | 2.69 | [M+H]^+^ | 245.0768 | 245.0771 | 1.18 | 113, 96, 70 | C |
| 14 | L-Tyrosine | C_9_H_11_NO_3_ | 2.90 | [M+H]^+^ | 182.0817 | 182.0807 | -5.49 | 136, 91 | C/O/A |
| 15 | L-Leucine | C_6_H_13_NO_2_ | 3.10 | [M+H]^+^ | 132.1025 | 132.1013 | -9.08 | 86, 44, 43 | A/O |
| 16 | Catalpol | C_15_H_22_O_10_ | 3.16 | [M+FA-H]^-^ | 407.1185 | 407.1195 | 2.46 | 199, 169, 151, 125, 97, 85, 44 | R |
| 17 | Adenosine | C_10_H_13_N_5_O_4_ | 3.44 | [M+H]^+^ | 268.1040 | 268.1036 | -1.60 | 136, 119 | C |
| 18 | Gallic acid* | C_7_H_6_O_5_ | 4.31 | [M-H]^-^ | 169.0143 | 169.0147 | 2.66 | 125, 97 | C |
| 19 | 5-hydroxymethyl-2-furfural | C_6_H_6_O_3_ | 4.39 | [M-H]^-^ | 125.0244 | 125.0247 | 2.40 | 125, 124, 96, 95, 79, 44, 41 | R |
| 20 | L-Phenylalanine | C_9_H_11_NO_2_ | 5.21 | [M+H]^+^ | 166.0868 | 166.0847 | -12.64 | 120, 103, 79, 77 | O |
| 21 | Rehmannioside D | C_27_H_42_O_20_ | 5.50 | [M+FA-H]^-^ | 731.2246 | 731.2247 | 0.14 | 505, 341, 323, 263, 221, 179 | R |
| 22 | Melittoside | C_21_H_32_O_15_ | 5.86 | [M+FA-H]^-^ | 569.1718 | 569.1705 | -2.28 | 181, 179, 161, 119, 89 | R |
| 23 | Ajugol* | C_15_H_24_O_9_ | 7.17 | [M+FA-H]^-^ | 393.1397 | 393.1397 | 0.00 | 347, 185, 167, 127, 44 | R |
|  |  |  |  | [M+NH_4_]^+^ | 366.1764 | 366.1751 | -3.55 | 169, 151, 123, 95 |  |
| 24 | Protocatechuic acid | C_7_H_6_O_4_ | 7.20 | [M-H]^-^ | 153.0193 | 153.0197 | 2.42 | 109, 108 | C |
| 25 | L-Tryptophan | C_11_H_12_N_2_O_2_ | 8.25 | [M+H]^+^ | 205.0977 | 205.0966 | -5.36 | 188, 146, 143, 118, 91 | O/G |
| 26 | Decaffeoyl-verbascoside | C_20_H_30_O_12_ | 8.26 | [M-H]^-^ | 461.1659 | 461.1657 | -0.43 | 315, 161, 152, 135, 113 | R |
| 27 | 8-Epiloganic acid | C_16_H_24_O_10_ | 8.28 | [M-H]^-^ | 375.1291 | 375.1286 | -1.33 | 213, 169, 151, 113, 95, 59 | R |
| 28 | Phloretic acid | C_9_H_10_O_3_ | 9.11 | [M-H]^-^ | 165.0557 | 165.0556 | -0.61 | 121, 93, 59, 41 | GR |
| 29 | 5-Hydroxyferulic Acid | C_10_H_10_O_5_ | 9.11 | [M-H]^-^ | 209.0455 | 209.0457 | 0.96 | 165, 121, 119, 59 | C |
| 30 | Glucoliquiritin | C_27_H_32_O_14_ | 9.73 | [M-H]- | 579.1719 | 579.1731 | 2.07 | 417, 255 | GR |
| 31 | Dihydroxycoumarin | C_9_H_6_O_4_ | 10.12 | [M-H]^-^ | 177.0188 | 177.02 | 6.78 | 177, 133, 105, 89 | GR |
| 32 | Nicotiflorin | C_27_H_30_O_15_ | 10.18 | [M-H]^-^ | 593.1512 | 593.152 | 1.35 | 503, 473, 383, 353 | J/GR |
|  |  |  |  | [M+H]^+^ | 595.1657 | 595.1658 | 0.17 | 541, 457, 409, 379, 325 |  |
| 33 | Purpureaside C/Echinacoside | C_35_H_46_O_20_ | 10.32 | [M-H]^-^ | 785.2504 | 785.2501 | -0.38 | 623, 161 | R |
| 34 | Dihydrocinnacasside | C_15_H_20_O_8_ | 10.52 | [M-H]^-^ | 327.1085 | 327.1092 | 2.02 | 165, 121 | C |
| 35 | Schaftoside* | C_26_H_28_O_14_ | 10.78 | [M-H]- | 563.1401 | 563.141 | 1.60 | 473, 443, 425, 353, 365, 323 | GR |
|  |  |  |  | [M+H]^+^ | 565.1557 | 565.156 | 0.53 | 481, 427, 325 |  |
| 36 | p-Coumaric acid glucoside | C_15_H_18_O_8_ | 10.85 | [M-H]^-^ | 325.0929 | 325.0939 | 3.08 | 163, 119 | C |
| 37 | Glucoisoliquiritin | C_27_H_32_O_14_ | 11.07 | [M-H]- | 579.1719 | 579.1732 | 2.24 | 255, 135 | GR |
| 38 | Licuraside | C_26_H_30_O_13_ | 11.55 | [M-H]^-^ | 549.1608 | 549.1621 | 2.37 | 429, 255, 135 | GR |
|  |  |  |  | [M+H]^+^ | 551.1765 | 551.1755 | -1.81 | 257, 137 |  |
| 39 | 4-Hydroxycinnamic acid | C_9_H_8_O_3_ | 11.67 | [M-H]^-^ | 163.0401 | 163.0409 | 5.09 | 119, 117, 93 | C |
| 40 | Rehmapicrogenin | C_10_H_16_O_3_ | 11.68 | [M-H]^-^ | 183.1021 | 183.1024 | 1.64 | 139, 123 | R |
| 41 | Liquiritin apioside* | C_26_H_30_O_13_ | 11.70 | [M-H]^-^ | 549.1614 | 549.1617 | 0.55 | 255, 135, 119, 91 | GR |
|  |  |  |  | [M+H]^+^ | 551.1759 | 551.1755 | -0.73 | 257, 137 |  |
| 42 | Neoliquiritin | C_21_H_22_O_9_ | 11.75 | [M-H]^-^ | 417.1191 | 417.1193 | 0.48 | 417, 255, 135, 119, 91 | GR |
|  |  |  |  | [M+H]^+^ | 419.1337 | 419.134 | 0.72 | 257, 147, 137 |  |
| 43 | Liquiritin* | C_21_H_22_O_9_ | 11.90 | [M-H]^-^ | 417.1191 | 417.1193 | 0.48 | 255, 135, 119, 91 | GR |
|  |  |  |  | [M+H]^+^ | 419.1337 | 419.134 | 0.72 | 257, 137 |  |
| 44 | 3,4-Dihydroxy-allylbenzene-4-O-α-L-rhamnopyranosyl(l→6)-β-D-glucopyranoside | C_21_H_30_O_11_ | 12.46 | [M-H]^-^ | 457.1724 | 457.1737 | 2.84 | 149 | O |
| 45 | quercetin 3-O-alpha-L-rhamnopyranosyl-(1->2)-alpha-L-arabinopyranoside | C_26_H_28_O_15_ | 12.47 | [M-H]^-^ | 579.1355 | 579.1363 | 1.38 | 579, 417, 300, 271, 181 | C |
| 46 | Ginsenoside Rh1 | C_36_H_62_O_9_ | 12.93 | [M+H]^+^ | 639.4472 | 639.4479 | 1.09 | 441, 423, 127 | G |
| 47 | Chalconaringenin 4-O-glucoside | C_21_H_22_O_10_ | 13.02 | [M-H]^-^ | 433.114 | 433.115 | 2.31 | 271, 177, 151, 119, 107 | GR |
| 48 | Apigenin | C_15_H_10_O_5_ | 13.05 | [M+H]^+^ | 271.0601 | 271.0603 | 0.74 | 271, 91 | GR |
| 49 | Tetrahydroxymethoxychalcone | C_16_H_14_O_6_ | 13.20 | [M-H]^-^ | 301.0712 | 301.0728 | 5.31 | 177, 150, 108 | GR |
| 50 | Ginsenoside Re* | C_48_H_82_O_18_ | 13.30 | [M+FA-H]^-^ | 991.5481 | 991.5513 | 3.23 | 945 | G |
| 51 | Isoliquiritin* | C_21_H_22_O_9_ | 13.67 | [M-H]^-^ | 417.1191 | 417.1189 | -0.48 | 417, 255, 135 | GR |
|  |  |  |  | [M+H]^+^ | 419.1337 | 419.1325 | -2.86 | 419, 257, 137 |  |
| 52 | 4-Hydroxybenzoic acid | C_7_H_6_O_3_ | 13.73 | [M-H]^-^ | 137.0244 | 137.0251 | 4.96 | 105, 93 | C |
| 53 | 7,5'-Dihydroxy-6,3'-dimethoxy-isoflavone-7-O-glucopyranoside | C_23_H_24_O_11_ | 13.75 | [M-H]- | 475.124 | 475.1243 | 0.63 | 267, 252 | GR |
| 54 | Ononin* | C_22_H_22_O_9_ | 13.76 | [M+H]^+^ | 431.1342 | 431.1322 | -4.64 | 269, 254 | GR |
| 55 | Licorice glycoside D2 | C_35_H_36_O_15_ | 13.81 | [M-H]^-^ | 695.1981 | 695.1998 | 2.45 | 549, 531, 255, 254, 145 | GR |
|  |  |  |  | [M+H]^+^ | 697.2127 | 697.2121 | -0.86 | 279, 257, 261, 147 |  |
| 56 | Neoisoliquiritin | C_21_H_22_O_9_ | 13.83 | [M-H]^-^ | 417.1191 | 417.1189 | -0.48 | 417, 255, 135, 119, 91 | GR |
|  |  |  |  | [M+H]^+^ | 419.1337 | 419.1325 | -2.86 | 257, 147, 137 |  |
| 57 | Wistin | C_23_H_24_O_10_ | 13.83 | [M+H]^+^ | 461.1442 | 461.1444 | 0.43 | 299, 284 | GR |
| 58 | Licorice glycoside C2* | C_36_H_38_O_16_ | 13.85 | [M-H]^-^ | 725.2087 | 725.2096 | 1.24 | 59, 531, 255, 193 | GR |
|  |  |  |  | [M+H]^+^ | 727.2233 | 727.224 | 0.96 | - |  |
| 59 | 4',7-Dihydroxyflavone/7,2'-dihydroxyflavone | C_15_H_10_O_4_ | 14.02 | [M+H]^+^ | 255.0657 | 255.0648 | -3.53 | 227, 145, 137, 119, 91 | GR |
| 60 | 24-Hydroxyl-licorice-saponin A3 | C_48_H_72_O_22_ | 14.23 | [M+H]^+^ | 1001.4588 | 1001.4624 | 3.59 | 839, 825, 663, 649, 631, 469, 451, 439, 423, 317 | GR |
| 61 | Licorice glycoside E | C_35_H_35_NO_14_ | 14.38 | [M-H]^-^ | 692.1985 | 692.2002 | 2.46 | 549, 531, 255, 160, 135 | GR |
|  |  |  |  | [M+H]^+^ | 694.213 | 694.2143 | 1.87 | - |  |
| 62 | Coumarin* | C_9_H_6_O_2_ | 14.40 | [M+H]^+^ | 147.0441 | 147.0434 | -4.49 | 103, 91, 77, 65 | C |
| 63 | Sydowic acid | C_15_H_20_O_4_ | 14.55 | [M-H]^-^ | 263.1289 | 263.1296 | 2.74 | 219, 204, 203, 151, 122 | C |
| 64 | Liquiritigenin* | C_15_H_12_O_4_ | 14.60 | [M-H]^-^ | 255.0663 | 255.0661 | -0.78 | 255, 135, 119, 91 | GR |
|  |  |  |  | [M+H]^+^ | 257.0808 | 257.0799 | -3.50 | 257, 239, 147, 137, 119, 109 |  |
| 65 | Licorice glycoside B | C_35_H_36_O_15_ | 14.62 | [M-H]^-^ | 695.1981 | 695.1998 | 2.45 | 549, 531, 255, 145 | GR |
| 66 | Uralsaponin F | C_44_H_64_O_19_ | 14.64 | [M+H]^+^ | 897.4115 | 897.4138 | 2.56 | 545, 527, 509, 175 | GR |
| 67 | Licoricesaponin A3 | C_48_H_72_O_21_ | 14.67 | [M-H]^-^ | 983.4488 | 983.449 | 0.20 | 821, 351 | GR |
|  |  |  |  | [M+H]^+^ | 985.4644 | 985.4657 | 1.32 | 809, 647, 615, 453, 471 |  |
| 68 | Azelaic acid | C_9_H_16_O_4_ | 14.69 | [M-H]^-^ | 187.0976 | 187.0975 | -0.53 | 187, 125, 123, 97, 95, 57 | C |
| 69 | 22-hydroxy-licoricesaponin G2 | C_42_H_62_O_18_ | 14.70 | [M-H]- | 853.3858 | 853.3863 | 0.59 | 351, 501 | GR |
|  |  |  |  | [M+H]^+^ | 855.4014 | 855.4024 | 1.17 | 661, 503, 485, 467, 261 |  |
| 70 | Calycosin* | C_16_H_12_O_5_ | 14.82 | [M-H]^-^ | 283.0612 | 283.062 | 2.83 | 268, 184, 148, 91 | GR |
|  |  |  |  | [M+H]^+^ | 285.0757 | 285.0759 | 0.70 | 270, 213, 137, 89 |  |
| 71 | Ginsenoside Rf* | C_42_H_72_O_14_ | 15.08 | [M-H]^-^ | 799.4844 | 799.4861 | 2.13 | 637, 475, 143 | G |
| 72 | Ginsenoside Rb1* | C_54_H_92_O_23_ | 15.08 | [M-H]^-^ | 1107.5951 | 1107.5941 | -0.90 | - | G |
|  |  |  |  | [M+NH_4_]^+^ | 1126.6373 | 1126.6370 | -0.27 | 487, 325 |  |
| 73 | 22β-Acetoxylglycyrrhizic acid | C_44_H_64_O_18_ | 15.13 | [M-H]- | 879.4014 | 879.4006 | -0.91 | 351 | GR |
|  |  |  |  | [M+H]^+^ | 881.4171 | 881.4173 | 0.23 | 529, 511 |  |
| 74 | Cinnamic acid | C_9_H_8_O_2_ | 15.22 | [M-H]^-^ | 147.0452 | 147.0460 | 5.78 | 103, 77, 61 | C |
|  |  |  |  | [M+H]^+^ | 149.0597 | 149.0595 | -1.41 | 103, 77 |  |
| 75 | 2-Hydroxycinnamaldehyde | C_9_H_8_O_2_ | 15.23 | [M+H]^+^ | 149.0597 | 149.0595 | -1.41 | 65, 77, 103, 131 | C |
| 76 | Ginsenoside Rg12 | C_42_H_72_O_15_ | 15.35 | [M-H]^-^ | 815.4793 | 815.4796 | 0.37 | 637, 475, 161 | G |
| 77 | Ginsenoside F3/Ginsenoside F5/Notoginsenoside R2 | C_41_H_70_O_13_ | 15.35 | [M-H]^-^ | 769.4738 | 769.4747 | 1.17 | 637, 475 | G |
| 78 | Uralsaponin E | C_42_H_60_O_17_ | 15.40 | [M-H]^-^ | 835.3752 | 835.3774 | 2.63 | 351 | GR |
|  |  |  |  | [M+H]^+^ | 837.3909 | 837.3924 | 1.79 | 485, 467, 449, 141 |  |
| 79 | Majonoside R1/isomer | C_42_H_72_O_15_ | 15.57 | [M-H]^-^ | 815.4793 | 815.4804 | 1.35 | 637, 161 | G |
| 80 | Echinatin | C_16_H_14_O_4_ | 15.63 | [M-H]^-^ | 269.0819 | 269.0828 | 3.34 | 237, 209, 183, 161,133, 120, 92 | GR |
|  |  |  |  | [M+H]^+^ | 271.0965 | 271.0967 | 0.74 | 229, 177, 134, 121, 107, 77 |  |
| 81 | Licoricesaponin M3 | C_48_H_70_O_20_ | 15.64 | [M-H]- | 965.4382 | 965.4399 | 1.76 | 497, 339 | GR |
| 82 | Naringenin* | C_15_H_12_O_5_ | 15.71 | [M-H]^-^ | 271.0612 | 271.0618 | 2.21 | 151, 119, 107, 93, 83 | GR |
| 83 | 3',4',7-Trihydroxyflavone | C_15_H_10_O_5_ | 15.76 | [M-H]^-^ | 269.0455 | 269.0467 | 4.46 | 135, 107 | GR |
|  |  |  |  | [M+H]^+^ | 271.0601 | 271.0603 | 0.74 | 215, 153, 91 |  |
| 84 | 6-Methylcoumarin/7-Methylcoumarin | C_10_H_8_O_2_ | 15.77 | [M+H]^+^ | 161.0597 | 161.0597 | -0.06 | 146, 118, 105, 90, 89, 77 | C |
| 85 | Licorice-saponin G2 or its isomer | C_42_H_62_O_17_ | 15.84 | [M-H]^-^ | 837.3914 | 837.3915 | 0.12 | 837, 351, 193 | GR |
|  |  |  |  | [M+H]^+^ | 839.406 | 839.4058 | -0.24 | 487， 469 |  |
| 86 | Licorice saponin E2 or isomer | C_42_H_60_O_16_ | 15.92 | [M-H]- | 819.3803 | 819.3814 | 1.34 | 351 | GR |
|  |  |  |  | [M+H]^+^ | 821.396 | 821.3981 | 2.56 | 451 |  |
| 87 | Pinellic acid | C_18_H_34_O_5_ | 16.01 | [M-H]^-^ | 329.2328 | 329.233 | 0.61 | 229, 211, 171, 139 | R |
| 88 | 22β-Acetoxyl licorice saponin C2 | _7_._00_ | 16.07 | [M-H]- | 863.4065 | 863.4077 | 1.39 | 601, 351, 89 | GR |
|  |  |  |  | [M+H]^+^ | 865.4222 | 865.4224 | 0.23 | 495 |  |
| 89 | Yunganoside G1 | C_48_H_74_O_21_ | 16.10 | [M-H]- | 985.4644 | 985.4662 | 1.83 | - | GR |
| 90 | Glycyrrhizic acid* | C_42_H_62_O_16_ | 16.32 | [M-H]^-^ | 821.3965 | 821.3981 | 1.95 | 821, 645, 469, 351, 194, 193 | GR |
|  |  |  |  | [M+H]^+^ | 823.4111 | 823.4105 | -0.73 | 347, 453 |  |
| 91 | Isoliquiritigenin* | C_15_H_12_O_4_ | 16.52 | [M-H]^-^ | 255.0663 | 255.0662 | -0.39 | 135, 119, 91 | GR |
|  |  |  |  | [M+H]^+^ | 257.0808 | 257.0799 | -3.50 | 257, 239, 147, 137, 119, 109 |  |
| 92 | Licoricesaponine B2 | C_42_H_64_O_15_ | 16.71 | [M-H]^-^ | 807.4167 | 807.4165 | -0.25 | 519, 351, 193 | GR |
|  |  |  |  | [M+H]^+^ | 809.4323 | 809.4329 | 0.74 | 633, 439, |  |
| 93 | 9,12,13,TriHODE | C_18_H_32_O_5_ | 16.76 | [M-H]^-^ | 327.2177 | 327.2185 | 2.44 | 239, 229, 171, 107 | C |
|  |  |  |  | [M+NH_4_]^+^ | 346.2588 | 346.2601 | 3.75 | 300, 256, 187 |  |
| 94 | Licorice saponin H2* | C_42_H_62_O_16_ | 16.81 | [M-H]- | 821.3965 | 821.3954 | -1.34 | 351 | GR |
|  |  |  |  | [M+H]^+^ | 823.4111 | 823.4105 | -0.73 | 647, 471, 453 |  |
| 95 | Formononetin* | C_16_H_12_O_4_ | 16.84 | [M-H]^-^ | 267.0663 | 267.0671 | 3.00 | 267, 252, 223, 195, 132, 91 | GR |
|  |  |  |  | [M+H]^+^ | 269.0808 | 269.0816 | 2.97 | 269, 253, 197, 118, 90 |  |
| 96 | 7-Methylcoumarin | C_10_H_8_O_2_ | 17.04 | [M+H]^+^ | 161.0597 | 161.0597 | 0.00 | 118, 105, 89, 77 | C |
| 97 | Truxillic Acid | C_18_H_16_O_4_ | 17.06 | [M-H]^-^ | 295.0976 | 295.0981 | 1.76 | 233, 147, 103, 96, 77 | C |
| 98 | Glycycoumarin | C_21_H_20_O_6_ | 17.13 | [M-H]^-^ | 367.1187 | 367.119 | 0.82 | 367, 309, 297, 284, 281, 265, 201 | GR |
|  |  |  |  | [M+H]^+^ | 369.1333 | 369.1345 | 3.25 | 369, 313, 301, 285, 270, 243, 227 |  |
| 99 | Uralsaponin C | C_42_H_64_O_16_ | 17.20 | [M+H]^+^ | 825.4273 | 825.4271 | -0.24 | 631, 455, 437, 409, 397, 353 | GR |
| 100 | Araboglycyrrhizin | C_41_H_62_O_14_ | 17.26 | [M-H]- | 777.4067 | 777.407 | 0.39 | 627 | GR |
| 101 | Licorice saponin C2 | C_42_H_62_O_15_ | 17.44 | [M-H]- | 805.4016 | 805.4025 | 1.12 | 351 | GR |
|  |  |  |  | [M+H]^+^ | 807.4161 | 807.417 | 1.11 | 437, 455 |  |
| 102 | Chikusetsusaponin Iva | C_42_H_66_O_14_ | 17.92 | [M-H]^-^ | 793.4374 | 793.4389 | 1.89 | 613, 569 | G |
| 103 | Isolicoflflavonol | C_20_H_18_O_6_ | 17.98 | [M+H]^+^ | 355.1176 | 355.1174 | -0.56 | 355, 299, 221, 153 | GR |
| 104 | 6-gingerol* | C_17_H_26_O_4_ | 17.98 | [M-H]^-^ | 293.1759 | 293.1757 | -0.68 | 57 | Z |
| 105 | Glicophenone | C_20_H_22_O_6_ | 18.07 | [M-H]^-^ | 357.1344 | 357.1353 | 2.52 | 247, 232, 189, 109 | GR |
|  |  |  |  | [M+H]^+^ | 359.1489 | 359.1494 | 1.39 | 193, 165, 137 |  |
| 106 | Licoarylcoumarin | C_21_H_20_O_6_ | 18.09 | [M-H]- | 367.1187 | 367.1187 | 0.00 | 309, 297 | GR |
|  |  |  |  | [M+H]^+^ | 369.1333 | 369.1338 | 1.35 | 285, 271 |  |
| 107 | Licoagroside A | C_23_H_24_O_12_ | 18.11 | [M+H]^+^ | 493.1346 | 493.1328 | -3.65 | 341, 313, 311, 285, 270, 243 | GR |
| 108 | Glyasperin C | C_21_H_24_O_5_ | 18.30 | [M+H]^+^ | 357.1697 | 357.1702 | 1.40 | 283, 165, 137, 123 | GR |
| 109 | 13-HPODE(1-) | C_18_H_32_O_4_ | 18.46 | [M-H]^-^ | 311.2228 | 311.2238 | 3.21 | 311, 223 | C |
| 110 | Ginsenoside Rg3* | C_42_H_72_O_13_ | 18.47 | [M-H]^-^ | 783.4895 | 783.4906 | 1.40 | 621, 161, 113 | G |
| 111 | Ginsenoside F4 | C_42_H_70_O_12_ | 18.50 | [M+H]^+^ | 767.4946 | 767.4950 | 0.52 | 443, 425, 407, 145, 99, 85 | G |
| 112 | Ophiopogonanone E | C_19_H_20_O_7_ | 18.52 | [M-H]^-^ | 359.1131 | 359.1165 | 9.47 | 344, 208, 207, 159, 154, 101 | O |
|  |  |  |  | [M+H]^+^ | 361.1287 | 361.1295 | 2.22 | 237, 137 |  |
| 113 | Ginsenoside Rg2 | C_42_H_72_O_13_ | 18.62 | [M-H]^-^ | 783.4895 | 783.4906 | 1.40 | 621, 161, 101 | G |
|  |  |  |  | [M+Na]^+^ | 807.4871 | 807.4962 | 11.27 | - |  |
| 114 | Ginsenoside Rg6 | C_42_H_70_O_12_ | 18.65 | [M+H]^+^ | 767.4946 | 767.4950 | 0.52 | 443, 425, 407, 145, 99, 85 | G |
| 115 | Licoisoflavone A | C_20_H_18_O_6_ | 18.81 | [M-H]^-^ | 353.1025 | 353.1031 | 1.70 | 284, 267, 243, 216, 211, 201, 174 | GR |
|  |  |  |  | [M+H]^+^ | 355.1182 | 355.1176 | -1.69 | 299, 191, 147 |  |
| 116 | Licoflavonol | C_20_H_18_O_6_ | 18.94 | [M+H]^+^ | 355.1176 | 355.1176 | 0.00 | 299, 217, 147, 91 | GR |
| 117 | Licochalcone A* | C_21_H_22_O_4_ | 18.96 | [M+H]^+^ | 339.1591 | 339.1592 | 0.29 | 121 | GR |
| 118 | Licoricone | C_22_H_22_O_6_ | 18.99 | [M-H]^-^ | 381.1344 | 381.1349 | 1.31 | 351, 323, 201, 109 | GR |
|  |  |  |  | [M+H]^+^ | 383.1489 | 383.1494 | 1.30 | 327, 299, 179, 149 |  |
| 119 | Licoflavone C | C_20_H_18_O_5_ | 19.18 | [M+H]^+^ | 339.1227 | 339.1232 | 1.47 | 283, 271, 181, 153 | GR |
| 120 | Glycyrol | C_21_H_18_O_6_ | 19.26 | [M-H]^-^ | 365.1031 | 365.1038 | 1.92 | 365, 307, 295, 282, 226, 183 | GR |
| 121 | Ophiopogonanone A* | C_18_H_16_O_6_ | 19.54 | [M-H]^-^ | 327.0869 | 327.0887 | 5.50 | 193, 192, 191, 164, 121 | O |
| 122 | Glabridin | C_20_H_20_O_4_ | 19.59 | [M-H]^-^ | 323.1283 | 323.1297 | 4.33 | 213, 201, 135, 91 | GR |
| 123 | Licoriphenone | C_21_H_24_O_6_ | 19.74 | [M-H]- | 371.15 | 371.151 | 2.69 | 261, 231, 109 | GR |
| 124 | l-(4-methyl-2-furanyl)-2-(5-methyl-5-ethenyl-2-tetrahydrofuranyl)-propan-l-one | C_15_H_20_O_3_ | 19.98 | [M+H]^+^ | 249.1491 | 249.1487 | -1.61 | 109, 93, 77, 55 | R |
| 125 | Glutinosalactone B | C_30_H_46_O_5_ | 19.99 | [M-H]^-^ | 485.3272 | 485.328 | 1.65 | 467, 423 | R |
| 126 | Licoisoflavone B | C_20_H_16_O_6_ | 20.08 | [M-H]^-^ | 351.0869 | 351.087 | 0.28 | 283, 241, 199, 83 | GR |
| 127 | Ginsenoside Rk1 | C_42_H_70_O_12_ | 20.34 | [M-H]^-^ | 765.4789 | 765.4799 | 1.31 | 603, 161 | G |
| 128 | Glabrol | C_25_H_28_O_4_ | 20.35 | [M-H]^-^ | 391.1909 | 391.1929 | 5.11 | 203, 187, 132 | GR |
| 129 | Methylophiopogonanone A | C_19_H_18_O_6_ | 20.39 | [M-H]^-^ | 341.1025 | 341.1038 | 3.81 | 207, 206, 178, 177 | O |
| 130 | 6-Shogaol* | C_17_H_24_O_3_ | 20.49 | [M+H]^+^ | 277.1798 | 277.1795 | -1.15 | 137, 155, 94, 77 | Z |
| 131 | Ginsenoside Rg5 | C_42_H_70_O_12_ | 20.54 | [M-H]^-^ | 765.4789 | 765.4800 | 1.44 | 603, 161 | G |
| 132 | Isoderrone | C_20_H_16_O_5_ | 20.55 | [M+H]^+^ | 337.1071 | 337.1074 | 0.89 | 321, 295, 153 | GR |
| 133 | Methylophiopogonanone B* | C_19_H_20_O_5_ | 20.66 | [M-H]^-^ | 327.1232 | 327.1246 | 4.28 | 206, 178, 163, 137 | O |
| 134 | Gancaonin E | C_25_H_28_O_6_ | 20.73 | [M-H]^-^ | 423.1813 | 423.1814 | 0.24 | 229, 203, 193, 149 | GR |
|  |  |  |  | [M+H]^+^ | 425.1959 | 425.1964 | 1.18 | 313, 175, 135 |  |
| 135 | Ceanothic acid | C_30_H_46_O_5_ | 20.78 | [M-H]^-^ | 485.3273 | 485.3280 | 1.55 | 467, 425, 423 | J |
| 136 | Glycyrdione A | C_25_H_28_O_5_ | 21.00 | [M-H]^-^ | 407.1864 | 407.1873 | 2.21 | 407, 219, 187 | GR |
| 137 | Isoangustone A/Glyurallin B | C_25_H_26_O_6_ | 21.26 | [M-H]^-^ | 421.1651 | 421.1662 | 2.61 | 366, 309, 217 | GR |
|  |  |  |  | [M+H]^+^ | 423.1808 | 423.1805 | -0.71 | 367, 311, 199,165 |  |
| 138 | Palmitic acid | C_16_H_32_O_2_ | 25.05 | [M-H]^-^ | 255.2330 | 255.2343 | 5.29 | - | C/CF |
| 139 | Ginsenoside Rg1 | C_42_H_72_O_14_ | 13.37 | [M-FA-H]^-^ | 845.4888 | 845.4895 | 0.83 | 799, 637, 161 | G |

GR: *Glycyrrhizae Radix Et Rhizoma Praeparata Cum Melle*; Z: *Zingiberis Rhizoma Recens*; G: *Ginseng Radix et Rhizoma*; R: *Rhemanniae Radix*; C: *Cinnamoni Ramulus*; A: *Asini Corii Colla*; O: *Ophiopogonis Radix*; CF: *Cannabis Fructus*; J: *Jujubae Fructus*. *Compared to reference standards.

**Supplementary Table 4.** Echocardiographic parameters of rats treated with ISO.

|  | Vehicle (n = 8) | ISO (n = 8) |
| --- | --- | --- |
| IVSd (mm) | 1.85 ± 0.15 | 2.69 ± 0.06^**^ |
| IVSs (mm) | 3.12 ± 0.10 | 4.30 ± 0.11^**^ |
| LVIDd (mm) | 5.99 ± 0.29 | 4.18 ± 0.29^**^ |
| LVIDs (mm) | 2.85 ± 0.14 | 1.41 ± 0.12^**^ |
| LVPWd (mm) | 2.74 ± 0.39 | 4.11 ± 0.30^*^ |
| LVPWs (mm) | 3.51 ± 0.31 | 4.61 ± 0.13^**^ |
| EF (%) | 82.70 ± 0.85 | 92.68 ± 1.49^**^ |
| FS (%) | 52.39 ± 0.95 | 65.94 ± 2.62^**^ |
| CO (mL/min) | 60.93 ± 8.05 | 26.71 ± 4.16^**^ |
| SV (μL) | 151.80 ± 16.80 | 75.97 ± 12.69^**^ |

IVS, interventricular septum; LVID, left ventricular internal diameter; LVPW, left ventricular posterior wall thickness; -d, diastolic; -s, systolic; EF, ejection fraction; FS, fractional shortening; CO, cardiac output; SV, stroke volume; Data are expressed as mean ± SEM. ^*^*P* < 0.05, ^**^*P* < 0.01 compared with the vehicle group.

**Supplementary Table 5.** ECG parameters of rats after HFLD treatment.

|  | Vehicle | ISO | HFLD_L_ | HFLD_M_ | HFLD_H_ | Met |
| --- | --- | --- | --- | --- | --- | --- |
| RR interval (ms) | 161.50 ± 6.10 | 173.70 ± 2.63 | 172.60 ± 4.70 | 165.80 ± 3.62 | 169.70 ± 3.49 | 165.00 ± 2.15 |
| PR interval (ms) | 49.32 ± 2.01 | 50.31 ± 2.09 | 50.97 ± 2.84 | 51.32 ± 1.51 | 52.35 ± 2.23 | 50.81 ± 1.27 |
| P wave (ms) | 13.68 ± 1.17 | 13.27 ± 0.92 | 12.45 ± 0.65 | 13.63 ± 0.99 | 13.77 ± 0.88 | 13.88 ± 0.98 |
| QRS (ms) | 18.01 ± 0.57 | 20.11 ± 2.17 | 17.21 ± 1.33 | 18.02 ± 1.86 | 16.06 ± 0.75 | 18.53 ± 0.79 |
| QTc (ms) | 113.00 ± 6.86 | 117.10 ± 6.13 | 107.30 ± 7.25 | 120.90 ± 4.28 | 120.00 ± 2.10 | 107.10 ± 5.75 |
| P wave amplitude (mV) | 0.060 ± 0.007 | 0.065 ± 0.007 | 0.064 ± 0.008 | 0.077 ± 0.009 | 0.066 ± 0.006 | 0.068 ± 0.004 |
| QRS amplitude (mV) | 0.55 ± 0.02 | 0.56 ± 0.04 | 0.58 ± 0.05 | 0.52 ± 0.07 | 0.58 ± 0.05 | 0.56 ± 0.06 |
| T wave amplitude (mV) | 0.041 ± 0.004 | 0.041 ± 0.006 | 0.040 ± 0.007 | 0.054 ± 0.008 | 0.056 ± 0.004 | 0.045 ± 0.010 |

n = 8. Data are expressed as mean ± SEM.

**Supplementary Table 6.** Echocardiographic parameters of rats after HFLD treatment.

|  | Vehicle | ISO | HFLD_L_ | HFLD_M_ | HFLD_H_ | Met |
| --- | --- | --- | --- | --- | --- | --- |
| IVSd (mm) | 1.80 ± 0.09 | 2.11 ± 0.07 | 2.07 ± 0.07 | 1.91 ± 0.11 | 1.95 ± 0.10 | 1.89 ± 0.08 |
| IVSs (mm) | 3.17 ± 0.12 | 3.41 ± 0.11 | 3.55 ± 0.09 | 3.30 ± 0.17 | 3.51 ± 0.17 | 3.32 ± 0.13 |
| LVIDd (mm) | 6.45 ± 0.25 | 6.41 ± 0.18 | 5.73 ± 0.21 | 6.24 ± 0.19 | 6.22 ± 0.15 | 6.36 ± 0.33 |
| LVIDs (mm) | 2.92 ± 0.18 | 3.47 ± 0.12 | 2.34 ± 0.24 | 2.79 ± 0.16 | 2.78 ± 0.16 | 2.77 ± 0.18 |
| LVPWd (mm) | 2.23 ± 0.28 | 2.73 ± 0.15 | 2.90 ± 0.21 | 2.64 ± 0.29 | 2.97 ± 0.26 | 2.77 ± 0.26 |
| LVPWs (mm) | 3.37 ± 0.20 | 3.27 ± 0.10 | 3.85 ± 0.15 | 3.62 ± 0.20 | 3.63 ± 0.13 | 3.81 ± 0.09 |
| EF (%) | 84.35 ± 1.59 | 76.18 ± 0.94^**^ | 87.92 ± 2.00^##^ | 85.11 ± 1.32^##^ | 84.98 ± 1.50^##^ | 85.85 ± 1.43^##^ |
| FS (%) | 54.79 ± 1.79 | 45.91 ± 0.86^*^ | 59.75 ± 3.05^##^ | 55.57 ± 1.62^#^ | 55.47 ± 1.84^#^ | 56.54 ± 1.71^##^ |
| CO (mL/min) | 66.92 ± 6.68 | 61.53 ± 3.40 | 55.07 ± 3.28 | 64.75 ± 3.45 | 63.92 ± 2.22 | 69.75 ± 9.74 |
| SV (μL) | 180.80 ± 14.32 | 160.40 ± 9.52 | 142.80 ± 9.45 | 168.40 ± 9.62 | 166.30 ± 7.48 | 181.20 ± 21.40 |

IVS, interventricular septum; LVID, left ventricular internal diameter; LVPW, left ventricular posterior wall thickness; -d, diastolic; -s, systolic; EF, ejection fraction; FS, fractional shortening; CO, cardiac output; SV, stroke volume; n = 8. Data are expressed as mean ± SEM. ^*^*P* < 0.05, ^**^*P* < 0.01 compared with the vehicle group. ^#^*P* < 0.05, ^##^*P* < 0.01 compared with the ISO group.
